# *Arhgef1* Deletion Disrupts Early Cortical Neurogenesis via PAK1 Overactivation and Causes Motor Dysfunction in Adulthood

**DOI:** 10.1002/advs.78426

**Published:** 2026-10-25

**Authors:** Liuren Li, Xuanyue Ma, Zhenyu Ding, Bang Sun, Linglin Liu, Hei Matthew Yip, Wei Wang, Tianying Wu, Yuan Guo, Yinghui Peng, Shiqing Zhang, Jacque Pak Kan Ip, Li Zhang, Keshen Li, Lei Shi

**Affiliations:** ^1^ State Key Laboratory of Bioactive Molecules and Druggability Assessment Guangdong Basic Research Center of Excelence for Natural Bioactive Molecules and Discovery of Innovative Drugs Jinan University Guangzhou China; ^2^ JNU‐HKUST Joint Laboratory for Neuroscience and Innovative Drug Research Guangdong Hong Kong‐Macau Joint Laboratory for Pharmacodynamic Constituents of TCM and New Drugs Research Jinan University Guangzhou China; ^3^ Department of Pharmacy Zhujiang Hospital Southern Medical University Guangzhou China; ^4^ Department of Pharmacy School of Medicine the Sixth Affiliated Hospital of South China University of Technology (Nanhai District People's Hospital of Foshan) Foshan China; ^5^ Guangdong‐Hong Kong‐Macau Institute of CNS Regeneration Key Laboratory of CNS Regeneration (Ministry of Education) Jinan University Guangzhou China; ^6^ School of Biomedical Sciences and Gerald Choa Neuroscience Institute The Chinese University of Hong Kong Hong Kong China; ^7^ Neurological Research Institute of Integrated Traditional Chinese and Western Medicine First School of Clinical Medicine The First Affiliated Hospital Guangdong Pharmaceutical University Guangzhou China

**Keywords:** ARHGEF1, motor function, neurodevelopmental disorder, neurogenesis, Rac1‐PAK1 signaling

## Abstract

Precise regulation of early cortical development is essential for the establishment of normal brain function, and its disruption can contribute to the pathogenesis of neurodevelopmental disorders (NDDs). Impaired motor function is a prominent early manifestation of various NDDs, yet the mechanisms remain elusive. Here, we identified ARHGEF1, a Rho family guanine nucleotide exchange factor highly expressed during early corticogenesis, as a novel regulator in embryonic cortical neurogenesis and adult motor function. By Emx1‐Cre‐mediated forebrain deletion of *Arhgef1* in mice (*Arhgef1* cKO mice), early cortical neurogenesis is specifically disrupted, leading to reduced deep‐layer cortical neurons. Mechanistically, *Arhgef1* deficiency overactivates RAC1/CDC42‐PAK1‐Cofilin signaling in early cortical development, whereas pharmacological PAK1 inhibition within this critical window rescues the genesis of deep‐layer cortical neurons. Importantly, *Arhgef1* cKO mice exhibit remarkable motor learning and coordination deficits in adulthood, accompanied by altered neuronal morphology in the motor cortex and impaired neuronal activities in motor circuits. In all, we elucidate a critical developmental window when ARHGEF1 governs corticogenesis via negatively regulates PAK1‐dependent pathway and further reveal an important role for ARHGEF1 in maintaining normal motor function in adulthood.

## Graphical abstract

ARHGEF1 plays a pivotal role in both embryonic cortical neurogenesis and adult motor function. During early development, it governs genesis of deep‐layer cortical neurons by negatively regulating RAC1/CDC42‐PAK1‐Cofilin signaling. In adulthood, ARHGEF1 is important for the maintenance of neuronal morphology and activity in the motor cortex, ensuring accurate motor circuitry and motor function.

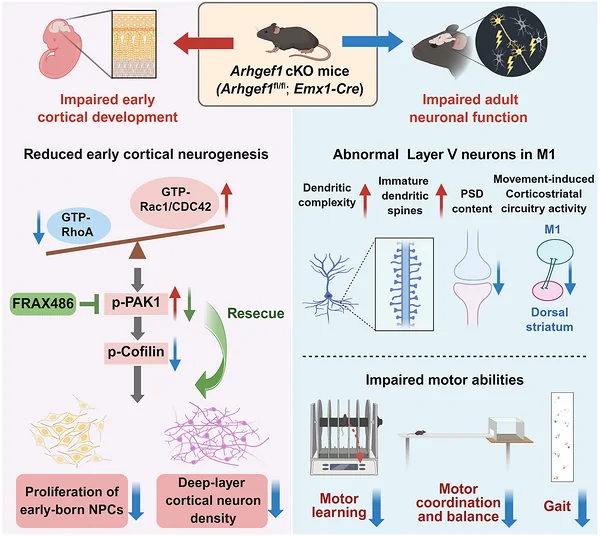

## 1 Introduction

The development of the cerebral cortex, which begins from the embryonic stage and continues into the early postnatal stage, is a highly coordinated and intricate sequence of processes that determines neural diversity, anatomical structure, and synaptic connectivity. Early cortical developmental processes, including progenitor cell proliferation, neurogenesis, neuronal migration, and differentiation, are essential for the establishment of the cellular composition and laminar organization of the cortex [1, 2]. These early events provide the structural basis for subsequent circuit formation and maturation, whereas their disruptions can cause persistent cortical dysfunctions that lead to cognitive, motor, social, and emotional deficits, contributing critically in the pathogenesis of neurodevelopmental disorders (NDDs) [3, 4, 5]. Importantly, delayed motor development and poor coordination are early‐onset, transdiagnostic features of various NDDs, including autism spectrum disorder (ASD) and attention deficit hyperactivity disorder (ADHD), and often serve as early indicators of broader developmental challenges [6, 7]. Nonetheless, the molecular mechanisms underlying early corticogenesis and motor circuit formation are not fully understood.

The highly dynamic changes of the actin cytoskeletal network play central roles in different cortical developmental events, including neural progenitor cell (NPC) proliferation, cell fate determination, neuronal migration and polarization, and dendritic spine morphogenesis [8, 9, 10, 11]. The dynamic balance between filamentous actin (F‐actin) and monomeric globular actin (G‐actin) is essential for proper neurodevelopment, while dysregulated homeostasis of them is strongly associated with NDDs, such as Fragile X syndrome (FXS) and developmental epilepsy [10, 12, 13]. Rho‐family GTPases, including Ras homolog gene family, member A (RHOA), Ras‐related C3 botulinum toxin substrate 1 (RAC1), and Cell division cycle 42 (CDC42), and their regulatory proteins, constitute a pivotal class of actin cytoskeleton regulators that govern various stages of cortical development [14, 15]. In particular, RAC1 and RHOA engage in a reciprocal negative feedback regulation mechanism, i.e., activated RHOA inhibits RAC1, whereas activated RAC1 stimulates its downstream effector protein p21‐activated kinase 1 (PAK1), which in turn inhibits RHOA [16, 17, 18]. The activated RAC1‐PAK1 induces a phosphorylation signaling cascade involving LIM kinase (LIMK) and Cofilin, transducing neural signals to actin reorganization [16, 17, 18]. Mutations of genes of *RAC* and *PAK* family, which result in either gain‐ or loss‐of function of RAC or PAK, are linked to brain malformations, intellectual disabilities, motor impairments, and epilepsy [19, 20, 21, 22, 23, 24]. Notably, the inhibition of PAK1 by genetic or pharmacological approaches corrects cellular abnormalities caused by RAC1‐PAK1 hyperactivation and shows great potential in alleviating NDD‐associated phenotypes in models of schizophrenia and FXS [25, 26, 27, 28]. However, whether the RAC‐PAK signaling pathway participates in the regulation of motor development remains unclear.

Rho GTPases act as molecular switches, shuttling between the active “on” state, stimulated by the guanine nucleotide exchange factors (GEFs), and the inactive “off” stage, catalyzed by the GTPase‐activating proteins (GAPs). Over one hundred of Rho GEFs and GAPs with distinct expression profiles across various developmental stages, cells, or subcellular structures have been identified to date, thereby forming a spatiotemporally specific regulatory network for Rho GTPases [29, 30]. Therefore, identifying key Rho GEFs/GAPs in specific developmental stages is important for the understanding of particular NDD‐related phenotypes. Rho guanine nucleotide exchange factor 1 (ARHGEF1), also known as p115 RhoGEF or Lsc, is prominently expressed in the embryonic brain and declines with development [31, 32]. It is highly expressed in NPCs and newborn neurons in the developing cortex, with its levels decreasing as neurons differentiate. The knockdown of *Arhgef1* leads to immature neurogenesis and excessive neurite outgrowth, suggesting an involvement in the checkpoint of transition from NPCs proliferation to neurogenesis [31, 32, 33]. Although ARHGEF1 is known as a RHOA GEF [34], the regulatory role of ARHGEF1 in neurogenesis appears to extend beyond RHOA, because an inhibitor of RhoA‐associated kinase (ROCK), an essential RHOA effector, could not block the effect of ARHGEF1 overexpression [31]. However, insights into the neuronal roles of ARHGEF1 have been derived solely from in vitro studies using cultured NPCs or neurons, whether ARHGEF1 serves as a critical determinant of cortical development and motor function remains unexplored.

In the present study, we generated a line of *Arhgef1* conditional knockout (cKO) mice by crossing *Arhgef1*‐floxed mice with *Emx1‐Cre* mice, depleting *Arhgef1* expression in the progenitors of forebrain excitatory neurons from early embryonic stages. Our findings revealed that *Arhgef1* deficiency resulted in reduced proliferation and increased immature cell cycle exit of early NPCs, leading to reduced neuron numbers in deep layers (Layers V–VI) of motor cortex. By contrast, neuron numbers from upper motor cortical layers (Layers II–IV) originating from late NPC pools were unaltered upon *Arhgef1* deficiency. We identified that decreased RHOA activity, but increased activity of the RAC1‐PAK1‐Cofilin signaling pathway, resulted in the cortex of *Arhgef1* cKO mice. Administration of the PAK1 inhibitor FRAX486 rescued early NPC pool size and subsequent deep layer neuron number. Notably, adult *Arhgef1* cKO mice showed reduced motor coordination and motor learning, accompanied by morphological and functional abnormalities of motor cortical neurons and impairment of motor cortical‐dorsal striatal circuits. Taken together, our results elucidate a pivotal role of ARHGEF1 in deep layer cortical neurogenesis by negatively regulating the RAC1‐PAK1 signaling pathway, and further reveal that ARHGEF1 is essential for motor circuit formation and motor functioning.

## 2 Results

### 2.1 Reduced Deep Layer Cortical Neurons in *Arhgef1* cKO Mice

Consistent with our previous report on ARHGEF1 protein levels [31, 32], cortical *Arhgef1* mRNA reduced sharply from embryonic day (E) 12.5 to E16.5, decreased further after birth, and reached its lowest level at postnatal day (P) 60 (Figure S1a). Hippocampal *Arhgef1* mRNA increased prenatally, peaked at P0, and decreased progressively from P0 to P60 (Figure S1b). These results suggest that ARHGEF1 is highly expressed in the embryonic forebrain, especially the early developing cortex. At early cortical developmental stages such as E13.5, ARHGEF1 is highly expressed in neural stem cells that positively stained sex determining region Y‐box 2 (SOX2) (Figure S1c,d). In adulthood, ARHGEF1 showed more colocalization with neurons, marked by Neuronal nuclei (NeuN), than with neuroglial cells (Figure S1e,f). Within the neurons, ARHGEF1 is expressed slightly more in excitatory neurons than inhibitory neurons (Figure S1g,h).

To dissect the role of ARHGEF1 in cortical development, we constructed a forebrain‐specific *Arhgef1* deletion (*Arhgef1* cKO) mouse line by crossing *Arhgef1‐*floxed mice with *Emx1*‐*Cre* mice (Figures 1a and S2a). We first verified the knockout efficiency of *Arhgef1* in the forebrain of cKO mice (Figure 1b–f). Both mRNA and protein levels of *Arhgef1* were reduced by approximately 50% in the cortex of *Arhgef1* cKO mice at E12.5 (Figure 1b,d,e). The reduction reached 75–85% in the adult cortex and hippocampus (Figure 1c,d,f). *Arhgef1* mRNA and protein were found unchanged in the cerebellum and thalamus of the cKO mice, validating that the deletion is limited to the forebrain (Figure S2b–d). *Arhgef1* cKO mice were grossly normal, having similar natality, growth rate, and brain weight with their wild‐type littermates (Figure S2e–h). However, by examining cortical lamination, we observed cell number alterations in the primary motor cortex (M1) and primary somatosensory cortex (SS1) of *Arhgef1* cKO mice at both P6 and adult age (Figure 1g). In particular, the numbers of deep‐layer neurons labeled by CTIP2 (Layer V) and TBR1 (Layer VI) decreased significantly in both M1 and SS1 of the cKO mice, whereas upper‐layer neurons labeled by CUX1 (Layer II–IV) showed no change (Figure 1h–k). The thickness of different cortical layers of M1 and SS1 remained unaltered in the cKO mice (Figure 1l–o). The hippocampal architecture of *Arhgef1* cKO mice was grossly normal, showing no difference in neuron numbers in the CA1 and CA3 regions when compared to those of WT littermates (Figure S2i–k). Therefore, forebrain deletion of *Arhgef1* mainly leads to reduced neuronal numbers specifically in the deep cortical layers, suggesting that ARHGEF1 is an indispensable factor during early developmental stages for cortical formation.

**FIGURE 1:**
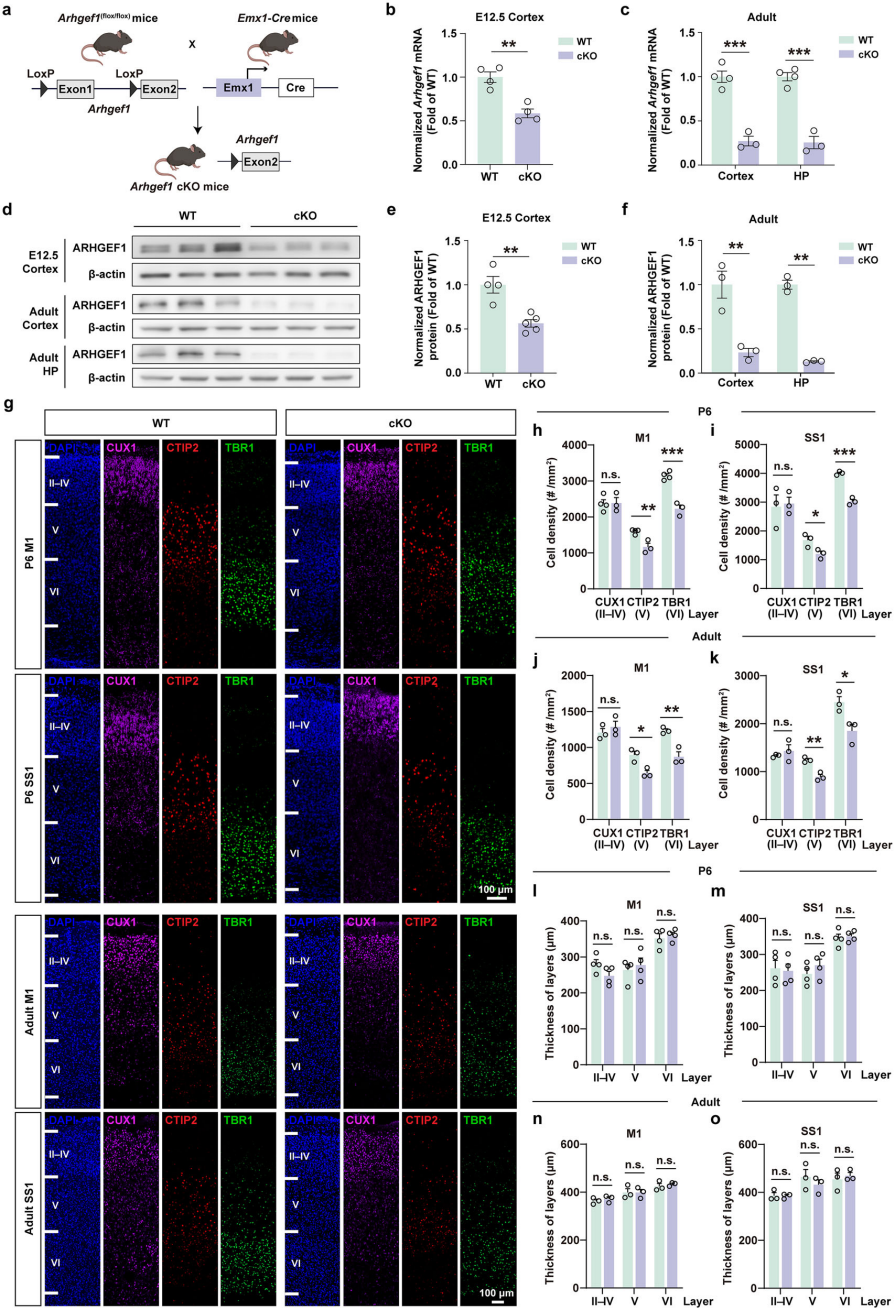
Reduced deep‐layer cortical neurons in *Arhgef1* cKO mice. (a) A schematic showing the Cre‐LoxP strategy for generation of *Arhgef1* cKO mice. (b) *Arhgef1* mRNA expression levels in the cortex of WT and cKO mice at E12.5. *n* = 4 mice per group. Unpaired *t*‐test, **p = 0.0019. (c) *Arhgef1* mRNA expression levels in the cortex and hippocampus of WT and cKO mice in adulthood. HP, hippocampus. WT, *n* = 4 mice, cKO, *n* = 3 mice. Unpaired *t*‐tests, cortex, ***p = 0.0004; HP, ***p = 0.0002. (d) Western blots of ARHGEF1 protein expression in the cortex of WT and cKO mice at E12.5, and in the cortex and hippocampus in adulthood. β‐actin serves as a loading control. (e) Quantification analysis of ARHGEF1 protein expression in the E12.5 cortex. WT, *n* = 4 mice, cKO, *n* = 5 mice. Unpaired *t*‐test, **p = 0.0026. (f) Quantification analysis of ARHGEF1 protein expression in adult cortex and hippocampus. *n* = 3 mice per group. Cortex, unpaired *t*‐test, **p = 0.0087; HP, Welch's *t*‐test, **p = 0.0029. (g) Immunofluorescence for DAPI and cortical layer markers (CUX1, violet; CTIP2, red; TBR1, green) in the primary motor cortex (M1) and primary somatosensory cortex (SS1) of WT and cKO mice at P6 and in adulthood. Layers are identified by DAPI‐stained nuclear distribution pattern. Scale bar, 100 µm. (h) Quantification of CUX1^+^ (Layers II–IV), CTIP2^+^ (Layer V), and TBR1^+^ (Layer VI) neuronal densities (#/mm^2^) in M1 at P6. WT, *n* = 4 mice, cKO, *n* = 3 mice. Unpaired *t*‐tests, CUX1, *p* = 0.9251; CTIP2, **p = 0.0062; TBR1, ***p = 0.0004. (i) Neuronal densities of different layers in SS1 at P6. *n* = 3 mice per group. Unpaired *t*‐tests, CUX1, *p* = 0.8345; CTIP2, *p = 0.0419; TBR1, ***p = 0.0003. (j) Neuronal densities of different layers in adult M1. *n* = 3 mice per group. Unpaired *t*‐tests, CUX1, *p* = 0.4427; CTIP2, *p = 0.0163; TBR1, **p = 0.0077. (k) Neuronal densities of different layers in adult SS1. *n* = 3 mice per group. Unpaired *t*‐tests, CUX1, *p* = 0.4892; CTIP2, **p = 0.004; TBR1, *p = 0.0313. (l) Thickness of different layers in M1 at P6. WT, *n* = 4 mice, cKO, *n* = 4 mice. Unpaired *t*‐tests, CUX1, *p* = 0.1107; CTIP2, *p* = 0.6069; TBR1, *p* = 0.5953. (m) Thickness of different layers in SS1 at P6. *n* = 4 mice per group. Unpaired *t*‐tests, CUX1, *p* = 0.8154; CTIP2, *p* = 0.3242; TBR1, *p* = 0.7202. (n) Thickness of different layers in adult M1. *n* = 3 mice per group. Unpaired *t*‐tests, CUX1, *p* = 0.4606; CTIP2, *p* = 0.9720; TBR1, *p* = 0.5047. (o) Thickness of different layers in adult SS1. *n* = 3 mice per group. Unpaired *t*‐tests, CUX1, *p* = 0.9031; CTIP2, *p* = 0.4421; TBR1, *p* = 0.6329. n.s., no significant difference. All data are shown as mean ± SEM.

### 2.2 Disrupted Proliferation of Neuronal Progenitor Cells During Early Cortical Development of *Arhgef1* cKO Mice

To elucidate the detailed roles of ARHGEF1 in early cortical development, we took advantage of the 5‐Ethynyl‐2'‐deoxyuridine (EdU) birthdating approach to separately investigate deep‐layer and upper‐layer neurogenesis. Newborn cortical progenitors in the embryos were pulse‐labeled by intraperitoneal (i.p.) injection of EdU (50 mg/kg) to pregnant *Arhgef1* cKO mice at E12.5–13.5 or E15.5–16.5, when Layer V–VI or Layer II–IV neurons are generated, respectively. The number of S‐phase proliferating cells over 1 h period of EdU incorporation at E13.5 or E16.5 was quantified. Compared to WT embryos, cKO embryos showed significantly reduced proliferation of cortical progenitors at E13.5 but not E16.5 (Figure 2a–d). Consistently, the number of cells labeled by Ki67, a marker of all active phases of the cell cycle, was also markedly reduced in the cKO group at E13.5 (Figure 2e–h). Moreover, cell cycle exit was assessed by quantifying EdU‐labeled cells that were devoid of Ki67 after 24 h period. Notably, more percentage of EdU‐birthdated cortical progenitors had exit from cell cycle after 24 h in the cKO embryos at E13.5 but not E16.5 (Figure 2e,f,i,j). Immunostaining of pH3 further revealed a reduction of M‐phase cells in cKO embryos at E13.5 but not E16.5 (Figure 2k–n).

**FIGURE 2:**
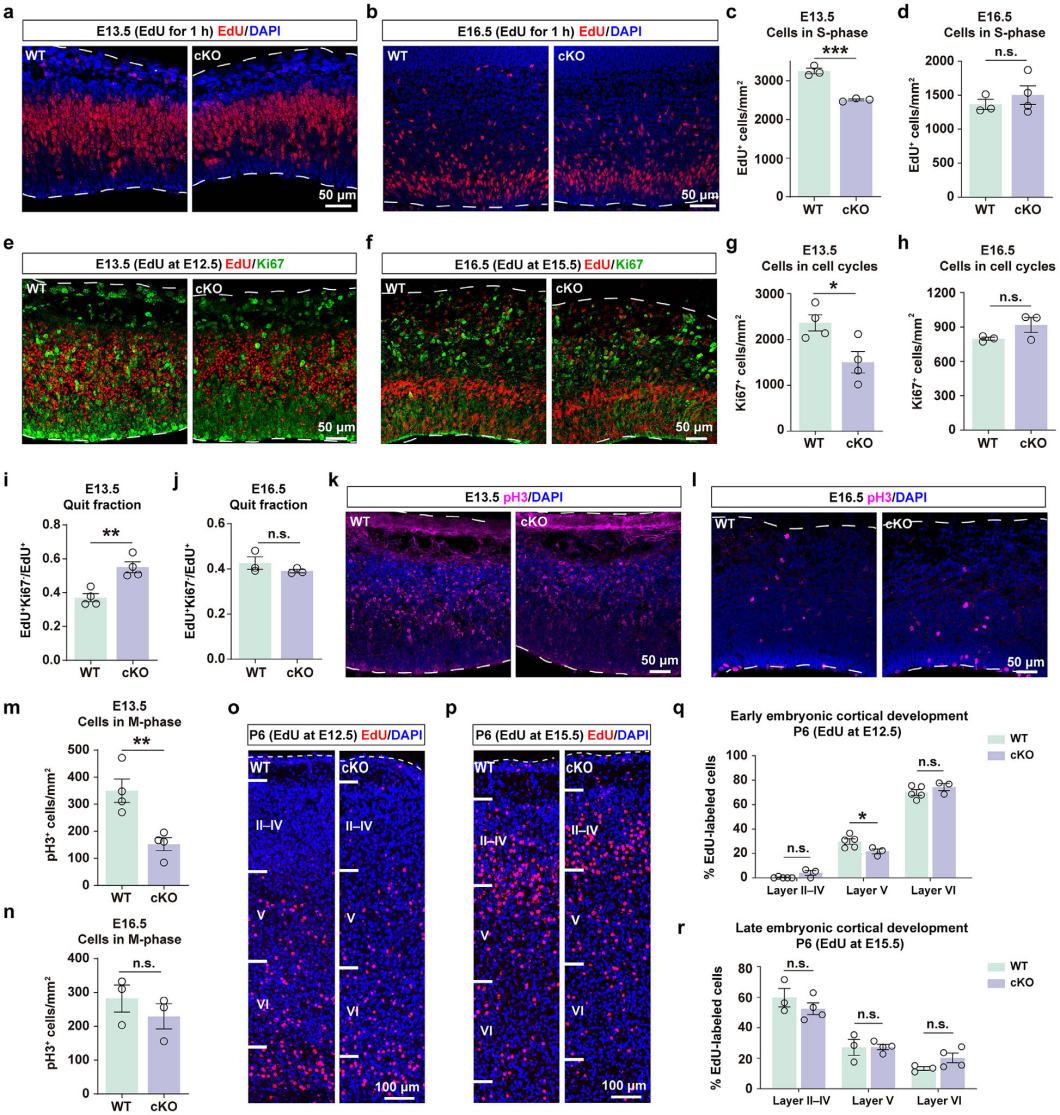
Disrupted proliferation of neuronal progenitor cells during early cortical development of *Arhgef1* cKO mice. (a, b) Representative cortical sections stained with EdU (red) and DAPI (blue) from WT and cKO mice at E13.5 (a) or E16.5 (b). Pregnant dams were intraperitoneally injected with EdU 1 h before embryo collection to label S‐phase cells. Scale bar, 50 µm. (c) Number of S‐phase cells (EdU^+^ cells) per mm^2^ in the cortex of WT and cKO mice at E13.5. *n* = 3 mice per group. Unpaired *t*‐test, ***p = 0.0007. (d) Number of S‐phase cells per mm^2^ at E16.5. WT, *n* = 3 mice, cKO, *n* = 4 mice. Unpaired *t*‐test, *p* = 0.4647. (e, f) Representative cortical sections with EdU (red) and Ki67 (green) from WT and cKO mice at E13.5 (e) or E16.5 (f). Pregnant dams were intraperitoneally injected with EdU 24 h before embryo harvest to label nascent cells. Scale bar, 50 µm. (g) Number of cycling (Ki67^+^) cells per mm^2^ in the cortex of WT and cKO mice at E13.5. *n* = 4 mice per group. Unpaired *t*‐test, *p = 0.0259. (h) Density of cycling cells at E16.5. *n* = 3 mice per group. Unpaired *t*‐test, *p* = 0.1459. (i) Quantification of cell cycle exit at E13.5. Quit fraction was calculated as (EdU^+^Ki67^−^ cells) / (total EdU^+^ cells). *n* = 4 mice per group. Unpaired *t*‐test, **p = 0.0041. (j) Quantification of cell cycle exit at E16.5. *n* = 3 mice per group. Unpaired *t*‐test, *p* = 0.2826. (k, l) Representative cortical sections stained with pH3 (violet) and DAPI (blue) from WT and cKO mice at E13.5 (k) or E16.5 (l). Scale bar, 50 µm. (m) Density of M‐phase (pH3^+^) cells in the cortex of WT and cKO mice at E13.5. *n* = 4 mice per group. Unpaired *t*‐test, **p = 0.0072. (n) Density of M‐phase cells at E16.5. *n* = 3 mice per group. Mann–Whitney test, *p* = 0.4000. (o, p) Representative cortical sections of WT and cKO mice stained with EdU (red) and DAPI (blue), showing laminar distribution of EdU‐pulse‐labeled cells birthdated at E12.5 (o) or E15.5 (p). Scale bar, 100 µm. (q) Proportion of E12.5‐birthdated EdU^+^ cells located within each cortical layer (II–IV, V, VI) was quantified at P6. WT, *n* = 5 mice, cKO, *n* = 3 mice. Unpaired *t*‐tests, layer II–IV, *p* = 0.0502; Layer V, *p = 0.0483; layer VI, *p* = 0.2815. (r) Proportion of E15.5‐birthdated EdU^+^ cells in each layer at P6. WT, *n* = 3 mice, cKO, *n* = 4 mice. Unpaired *t*‐tests, Layer II–IV, *p* = 0.1704; Layer V, *p* = 0.3215; Layer VI, *p* = 0.1142. n.s., no significant difference. All data are shown as mean ± SEM.

To examine the final laminar distribution and positioning of early‐ and late‐born neurons, we performed birthdating analysis by EdU injection at E12.5 or E16.5 and analyzed the distribution of labeled cells at P6, when all neurons have settled in their destined layers (Figure 2o–r). Early wave of neurons born from E12.5‐progenitor cells were mainly distributed to the deep cortical layers in both WT and cKO mice, but the layer V neuron number was remarkably fewer in the cKO group than in WT littermates (Figure 2o, q). By contrast, the late wave of neurons born from E15.5‐progenitor cells were mainly distributed to the upper cortical layers, without showing differences between the WT and cKO groups (Figure 2p,r). These findings collectively suggest that deletion of *Arhgef1* in neural progenitors specifically influences early cortical development, resulting in diminished proliferation but accelerated cell cycle exit of early progenitors, leading to a smaller progenitor pool size for the genesis of deep‐layer neurons.

### 2.3 Overactivation of RAC1‐PAK1‐Cofilin Signaling in Early Cortical Development of *Arhgef1* cKO Mice

To investigate the molecular mechanism accounted for the neurodevelopmental defects in *Arhgef1* cKO mice, we examined the activity changes of RHOA, a target known to be directly activated by ARHGEF1, along development. Consistent with the observation that ARHGEF1 levels peak in the early developmental cortex, RHOA activity was prominently reduced in the cKO cortex at E14.5 (Figure 3a,b). However, RHOA activity became comparable between the cKO and WT cortex at P6 and adult age, when ARHGEF1 levels were low (Figure 3a,b). Interestingly, RAC1 and CDC42, two other crucial Rho GTPases, were more active in the cKO cortex than in the WT cortex at E14.5, exhibiting opposite activity changes to that of RHOA (Figure 3a,c,d). The exaggerated activities of RAC1 and CDC42 were still observed to a lesser extent in the cKO cortex at P6. In adulthood, no differences in RAC1 and CDC42 activities were found in the cortex between cKO and WT mice (Figure 3a,c,d). Therefore, deletion of *Arhgef1* results in unbalanced changes of RHOA and RAC1/CDC42 in the developing cortex. Because ARHGEF1 is incapable of directly modulating RAC1 and CDC42 [34], changes in these two GTPases may be events induced by RHOA inactivation in the cKO cortex, which falls in line with the evidence that RHOA negatively regulates RAC1/CDC42. These results are consistent with our previous studies that ARHGEF1 increases RHOA activity but decreases RAC1 and CDC42 activities in Neuro‐2a cells [31].

**FIGURE 3:**
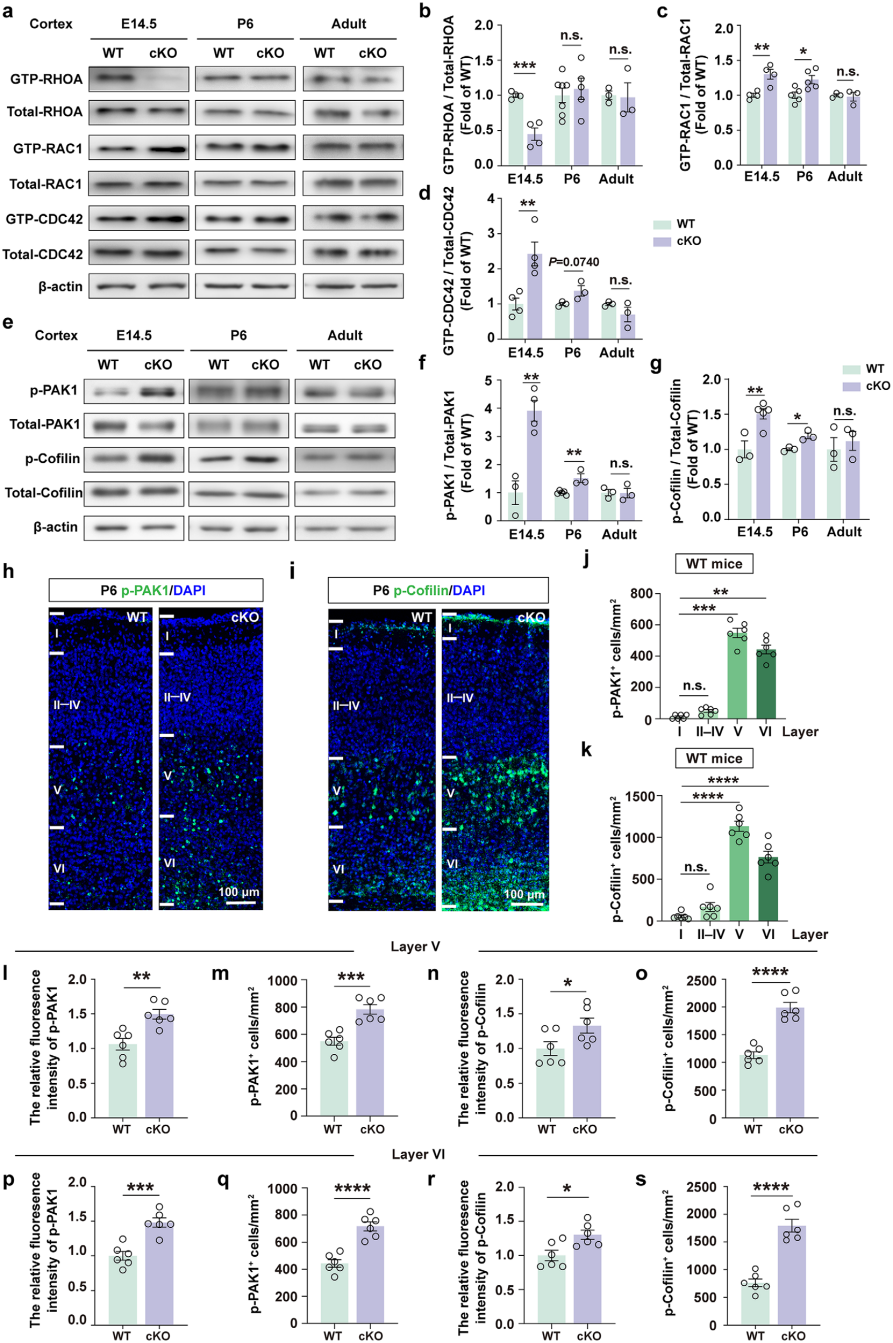
Overactivation of RAC1‐PAK1‐Cofilin signaling in early cortical development of *Arhgef1* cKO mice. (a) Representative Western blots showing GTP‐ and total‐protein levels of RHOA, RAC1, and CDC42 in the cortex of WT and cKO mice at E14.5, P6, and in adulthood. β‐actin serves as a loading control. (b) Quantification of GTP‐RHOA expression levels that were normalized to total‐RHOA. Unpaired *t*‐tests, E14.5, *n* = 4 mice per group, ***p = 0.0010; P6, WT, *n* = 7 mice, cKO, *n* = 5 mice, *p* = 0.6077; Adult, *n* = 3 mice per group, *p* = 0.9050. (c) Quantification of GTP‐RAC1 expression levels that were normalized to total‐RAC1. Unpaired *t*‐tests, E14.5, *n* = 4 mice per group, **p = 0.0075; P6, *n* = 5 mice per group, *p = 0.0181; Adult, *n* = 3 mice per group, *p* = 0.7656. (d) Quantification of GTP‐CDC42 expression levels that were normalized to total‐CDC42. Unpaired *t*‐tests, E14.5, *n* = 4 mice per group, **p = 0.0093; P6, *n* = 3 mice per group, *p* = 0.0740; Adult, *n* = 3 mice per group, *p* = 0.4555. (e) Representative Western blots showing phospho‐ (p) and total‐protein levels of PAK1, LIMK1, and Cofilin in the cortex of WT and cKO mice at E14.5, P6, and in adulthood. β‐actin serves as a loading control. (f) Quantification of p‐PAK1 levels that were normalized to total‐PAK1. Unpaired *t*‐tests, E14.5, WT, *n* = 3 mice, cKO, *n* = 4 mice, **p = 0.0034; P6, WT, *n* = 5 mice, cKO, *n* = 3 mice, **p = 0.0056; Adult, *n* = 3 mice per group, *p* = 0.9520. (g) Quantification of p‐Cofilin levels that were normalized to total Cofilin. Unpaired *t*‐tests, E14.5, WT, *n* = 3 mice, cKO, *n* = 5 mice, **p = 0.0053; P6, *n* = 3 mice per group, *p = 0.0122; Adult, *n* = 3 mice per group, *p* = 0.6077. (h, i) Representative cortical sections of WT and cKO mice immunostained for p‐PAK1 (h) or p‐Cofilin (i) (green) and DAPI (blue) at P6. Layers are outlined based on cellular density. (j) Number of p‐PAK1^+^ cells per mm^2^ in different layers from WT cortex. *n* = 6 slices from 3 mice. Kruskal–Wallis test with Dunn's multiple comparisons test, I vs. II–IV, *p* = 0.6608; I vs. V, ***p = 0.0001; I vs. VI, **p = 0.0086. (k) Density of p‐Cofilin^+^ cells in different layers from WT cortex. *n* = 6 slices from 3 mice. One‐way ANOVA with Bonferroni's multiple comparison test, I vs. II–IV, *p* = 0.4864; I vs. V, ****p < 0.0001; I vs. VI, ****p < 0.0001. (l) Mean fluorescent intensity of p‐PAK1 in Layer V from WT and cKO cortex. *n* = 6 slices from 3 mice in each group. Unpaired *t*‐test, **p = 0.0025. (m) Density of p‐PAK1^+^ cells in Layer V of WT and cKO cortex. *n* = 6 slices from 3 mice in each group. Unpaired *t*‐test, ***p = 0.0005. (n) Mean fluorescent intensity of p‐Cofilin in Layer V from WT and cKO cortex. *n* = 6 slices from 3 mice in each group. Unpaired *t*‐test, *p = 0.0466. (o) Density of p‐Cofilin^+^ cells in Layer V from WT and cKO cortex. *n* = 6 slices from 3 mice in each group. Unpaired *t*‐test, ****p < 0.0001. (p) Mean fluorescent intensity of p‐PAK1 in Layer VI from WT and cKO cortex. *n* = 6 slices from 3 mice in each group. Unpaired *t*‐test, ***p = 0.0004. (q) Density of p‐PAK1^+^ cells in Layer VI from WT and cKO cortex. *n* = 6 slices from 3 mice in each group. Unpaired *t*‐test, ****p < 0.0001. (r) Mean fluorescent intensity of p‐Cofilin in Layer VI from WT and cKO cortex. *n* = 6 slices from 3 mice in each group. Unpaired *t*‐test, *p = 0.0143. (s) Density of p‐Cofilin^+^ cells in Layer VI from WT and cKO cortex. *n* = 6 slices from 3 mice in each group. Unpaired *t*‐test, ****p < 0.0001. n.s., no significant difference. All data are shown as mean ± SEM.

Both RAC1/CDC42 and RHOA regulate the PAK1‐Cofilin signaling pathway, but in opposite ways. Thus, we examined this downstream signaling pathway to dissect which GTPase dominates the changes in early cortical development of *Arhgef1* cKO mice. Similar to the profile of RAC1 and CDC42, the phosphorylation levels of PAK1 and Cofilin were drastically upregulated at E14.5, slightly upregulated at P6, and displayed no changes in the adult cortex of cKO mice when compared to those of WT littermates (Figure 3e–g). In line with the fact that phosphorylation of Cofilin inhibits its actin‐depolymerizing effect, the filament (F)‐actin, labeled by phalloidin, was increased in the cortical plate and intermediate zone of cKO mice at E13.5 (Figure S3a,b). Unlike observations in the cortex, RHOA activity was lowered, but RAC1 and CDC42 activities were unchanged in the hippocampus of cKO mice at P6, and RAC1 was inhibited, but RHOA and CDC42 activities showed no changes in the adult hippocampus of cKO mice (Figure S4a–d). These results unveiled an exaggerated RAC1/CDC42‐PAK1‐Cofilin pathway specifically in early cortical development caused by *Arhgef1* deletion. To obtain the layer‐specific changes of activated PAK1 and Cofilin, we examined the laminal distribution of p‐PAK1 and p‐Cofilin immunosignals in cortical sections from cKO and WT mice at P6. Notably, both p‐PAK1 and p‐Cofilin were highly enriched in Layer V and VI in the WT cortex (Figure 3h–k), and the immunoreactive cell numbers and immunosignal intensities of p‐PAK1 and p‐Cofilin were both markedly increased in the deep layers of cKO cortex (Figure 3h,i,l–s). Interestingly, p‐PAK1 at E16.5 was significantly elevated in the marginal zone, cortical plate, subplate, and intermediate zone, but not in the subventricular/ventricular zone, of the cKO mice cortex (Figure S3c–e), suggesting that PAK1 hyperactivation is prominent in postmitotic neurons, including the early‐born subplate and deep‐layer neurons, at this embryonic stage. Taken together, these results showed that the RAC1/CDC42‐PAK1‐Cofilin pathway is overactivated in early cortical development that mainly affects deep‐layer cortical neurons in *Arhgef1* cKO mice.

### 2.4 PAK1 Inhibition at Early Cortical Development Corrects Abnormal Neurogenesis of Deep‐layer Cortical Neurons in *Arhgef1* cKO Mice

We next investigated whether the reduction of early NPC proliferation and neurogenesis is attributed to overactivation of the RAC1/CDC42‐PAK1‐Cofilin pathway in *Arhgef1* cKO mice. FRAX486 (10 mg/kg), a specific PAK1 inhibitor with potency of in vivo PAK1 inhibition that holds beneficial effects for alleviating neurodevelopmental disorder‐like behaviors [25, 28], was used for in utero PAK1 inhibition. FRAX486 or vehicle was i.p. administered to pregnant cKO mice at E12.5 that were mated with WT male mice, and the cortical tissues of the WT or cKO embryos were harvested at E14.5 to examine the PAK1‐Cofilin signaling pathway (Figure 4a). While vehicle‐administered cKO embryos still showed upregulated p‐PAK1 and p‐Cofilin in the cortex compared to WT littermates, FRAX486 administration reversed the overactivation of PAK1 and Cofilin, resulting in comparable activities of the two proteins between cKO and WT cortex (Figure 4b–d).

**FIGURE 4:**
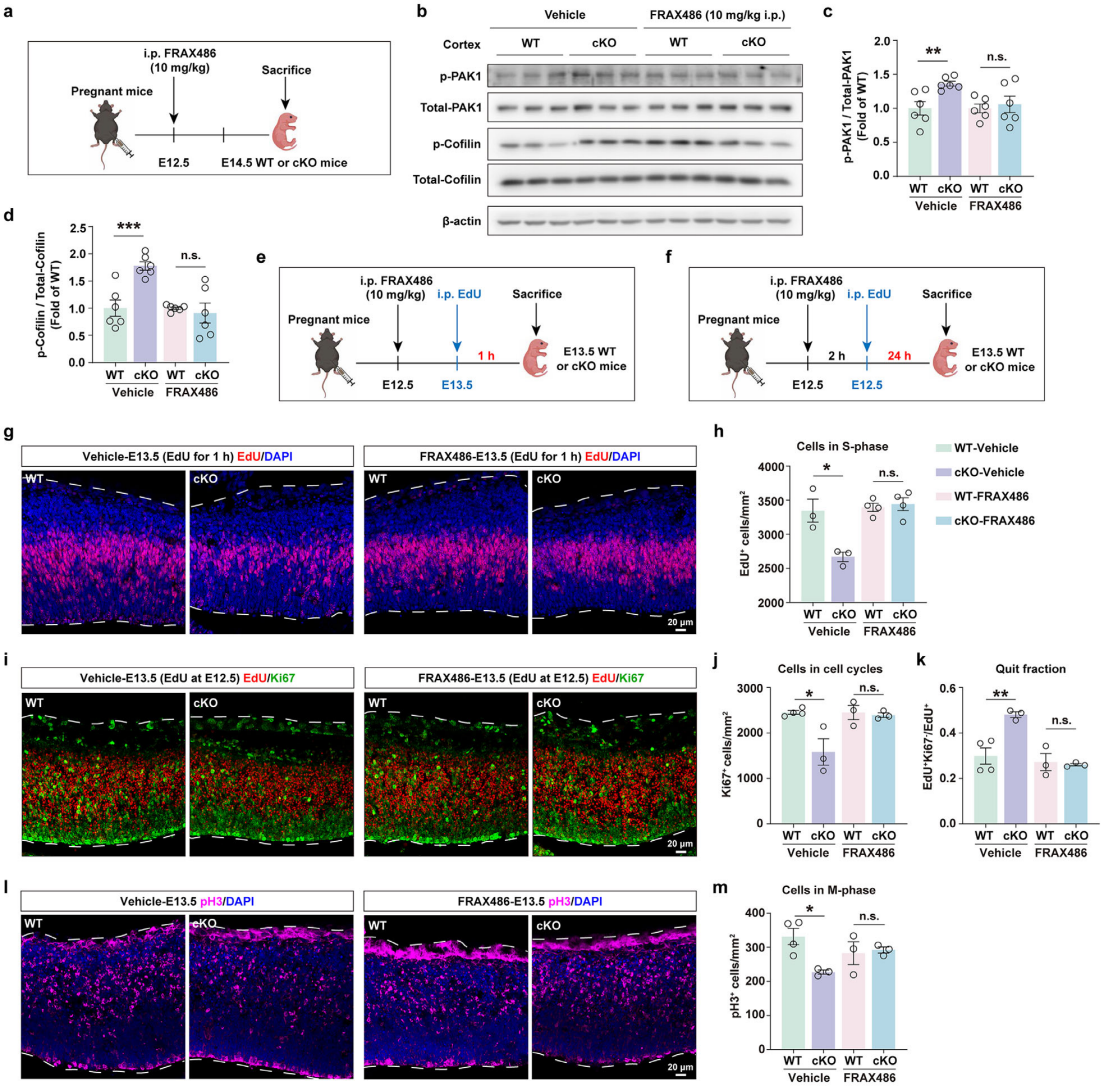
PAK1 inhibition at early cortical development corrects abnormal deep‐layer cortical neurogenesis in *Arhgef1* cKO mice. (a) Schematic of in utero FRAX486 (a PAK1 inhibitor) treatment strategy to inhibit hyperactivated PAK1‐Cofilin signaling in E12.5–14.5 cKO embryos. Pregnant dams were intraperitoneally injected with FRAX486 (10 mg/kg) at E12.5, followed by embryo harvest at E14.5. (b) Representative Western blots of p‐ and total protein levels of PAK1 and Cofilin in the cortex of WT and cKO mice after FRAX486 or vehicle treatment. β‐actin serves as a loading control. (c) Quantification of p‐PAK1 levels that were normalized to total‐PAK1. *n* = 6 mice per group. Unpaired *t*‐tests, Vehicle‐WT vs. Vehicle‐cKO, **p = 0.0061; FRAX486‐WT vs. FRAX486‐cKO, *p* = 0.6737. (d) Quantification of p‐Cofilin levels that were normalized to total‐Cofilin. *n* = 6 mice per group. Unpaired *t*‐tests, Vehicle‐WT vs. Vehicle‐cKO, ***p = 0.0010; FRAX486‐WT vs. FRAX486‐cKO, *p* = 0.6399. (e, f) Schematic of PAK1‐targeted rescue strategies for neuronal proliferation deficits in cKO mice. (e) Pregnant dams were treated with FRAX486 at E12.5 followed by EdU injection at E13.5, 1 h before embryo collection to label S‐phase cells. (f) Pregnant dams were treated with FRAX486 at E12.5 and then with EdU 2 h later, 24 h before embryo collection to label nascent cells. (g) Representative images obtained with the strategy in (e), showing EdU (red) and DAPI (blue) staining in the cortex of WT and cKO mice treated with FRAX486 or vehicle. Scale bar, 20 µm. (h) Density of S‐phase (EdU^+^) cells in different groups. Unpaired *t*‐tests, Vehicle‐WT (*n* = 3 mice) vs. Vehicle‐cKO (*n* = 3 mice), *p = 0.0205; FRAX486‐WT (*n* = 4 mice) vs. FRAX486‐cKO (*n* = 4 mice), *p* = 0.6926. (i) Representative images obtained with the strategy in (f), showing EdU (red) and Ki67 (green) staining. Scale bar, 20 µm. (j) Number of cycling (Ki67^+^) cells per mm^2^ in different groups. *n* = 4 mice per group. Unpaired *t*‐tests, *p = 0.0259. (k) Quantification of cell cycle exit in different groups. Quit fraction was calculated by (EdU^+^Ki67^−^ cells) / (total EdU^+^ cells). Unpaired *t*‐tests, Vehicle‐WT (*n* = 4 mice) vs. Vehicle‐cKO (*n* = 3 mice), **p = 0.0086; FRAX486‐WT (*n* = 3 mice) vs. FRAX486‐cKO (*n* = 3 mice), *p* = 0.7720. (l) Staining of pH3 (violet) and DAPI (blue) in the cortex of WT and cKO mice at E13.5, after treatment with FRAX486 or vehicle at E12.5. Scale bar, 20 µm. (m) Density of M‐phase (pH3^+^) cells in different groups. Unpaired *t*‐tests, Vehicle‐WT (*n* = 4 mice) vs. Vehicle‐cKO (*n* = 3 mice), *p = 0.0176; FRAX486‐WT (*n* = 3 mice) vs. FRAX486‐cKO (*n* = 3 mice), *p* = 0.8036. n.s., no significant difference. All data are shown as mean ± SEM.

Having confirmed that single injection of FRAX486 at E12.5 is sufficient to normalize PAK1 signaling up to E14.5, we designed two experiments to investigate the neurodevelopmental processes of cKO cortex after FRAX486 administration (Figure 4e,f). In the first experimental setting, FRAX486 was injected once at E12.5, and EdU (50 mg/kg) was injected to label nascent NPCs at E13.5. The embryonic brains were collected 1 h after EdU injection to measure NPC proliferation (Figure 4e). While vehicle‐administered cKO cortex had significantly fewer S‐phase EdU^+^ cells than the WT group, FRAX486 restored normal proliferation in the cKO cortex (Figure 4g,h). In another experimental setting, EdU was injected 2 h after FRAX486 administration at E12.5, and the cell cycle quitting fractions were analyzed at E13.5 (Figure 4f). Consistently, the number of Ki67⁺ cells was markedly reduced in the vehicle‐treated cKO group, and FRAX486 restored it to normal levels (Figure 4i,j). Moreover, the increased cell cycle quitting rate of progenitors in the cKO cortex was normalized by FRAX486 (Figure 4i,k). Consistently, the number of pH3‐labeled M‐phase cells decreased in the cKO relative to WT embryos after vehicle treatment, but showed no change between cKO and WT embryos after FRAX486 treatment (Figure 4l,m). We then investigated whether PAK1 inhibition during the embryonic neurogenesis window has a long‐term effect on cortical lamination. To this end, nascent cells were labeled by EdU at E12.5, and FRAX486 or vehicle was administered at E12.5, E14.5, and E17.5. This multi‐dose regimen was chosen because p‐PAK1 remained elevated in cKO cortex at E16.5 (Figure S3c–e), and similar repeated administration of FRAX486 exhibits notable rescuing effects in neurodevelopmental disorder models [27, 28]. The final positioning of these EdU‐labeled cells was examined at P6 (Figure 5a). As reported earlier, the Layer V EdU^+^ cells were fewer in the vehicle‐treated cKO pups, but FRAX486 corrected this abnormal phenotype (Figure 5b,c). Moreover, the decreased numbers of CTIP2^+^ neurons and TBR1^+^ neurons in the cKO mice were also restored by FRAX486 treatment (Figure 5d–f). Taken together, these results suggest that in utero normalization of PAK1 activity in early development restores neurogenesis of deep‐layer cortical neurons in *Arhgef1* cKO mice.

**FIGURE 5:**
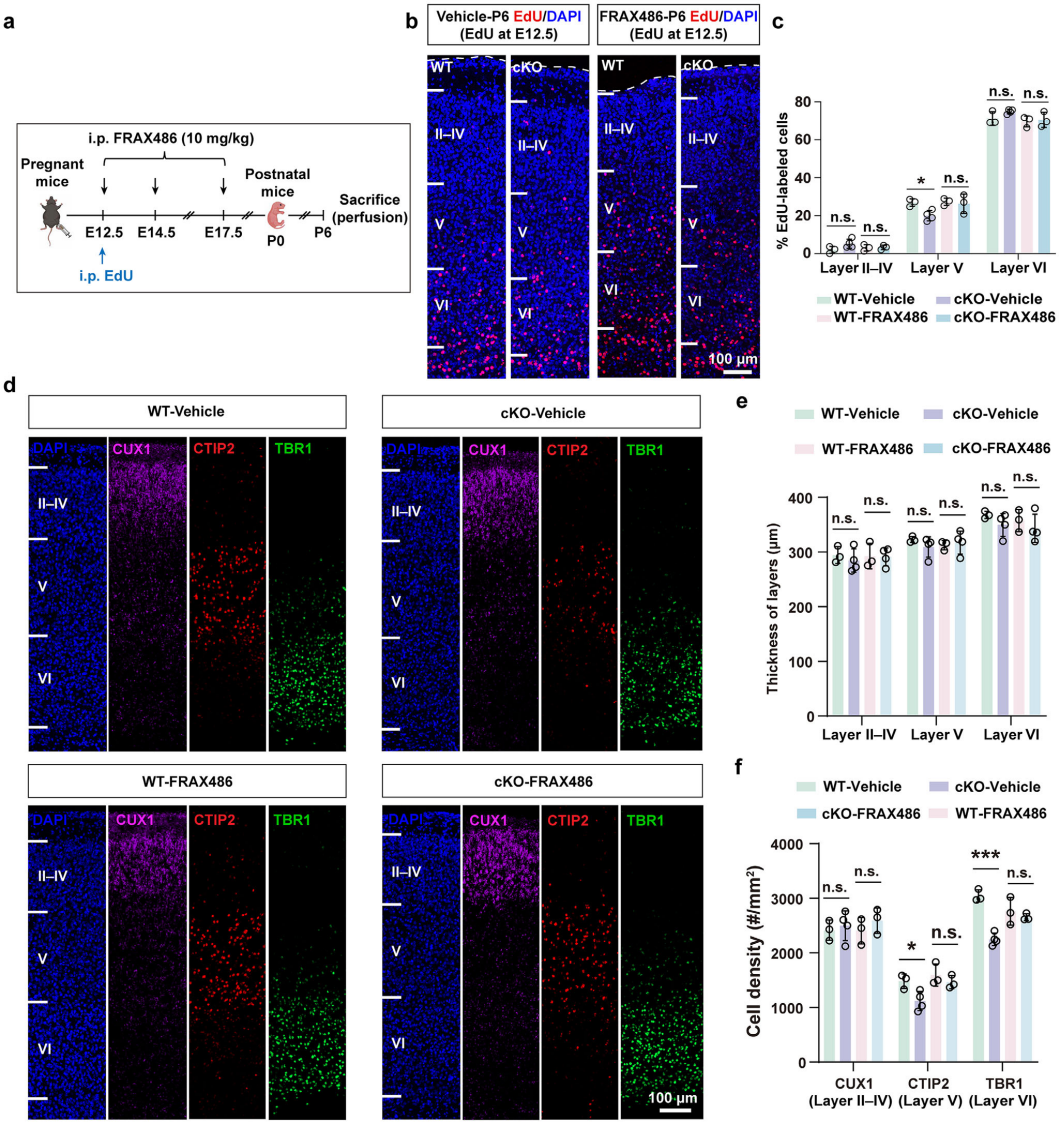
Inhibition of hyperactivated PAK1 during early cortical development rescues lamination defects in *Arhgef1* cKO mice. (a) Schematic of the PAK1‐targeted rescue strategy for cortical lamination defects in cKO mice. Pregnant mice were administered FRAX486 (10 mg/kg) at E12.5, E14.5, and E17.5 before embryo harvest at P6. (b) Staining of EdU (red) and DAPI (blue) in the cortex of WT and cKO mice at P6, with FRAX486 or vehicle treatment, showing laminal distribution of E12.5‐birthdated cells. Scale bar, 100 µm. (c) Proportion of E12.5‐birthdated EdU^+^ cells in each layer (II–IV, V, VI). Unpaired *t*‐tests, Layer II–IV, Vehicle‐WT (*n* = 3 mice) vs. Vehicle‐cKO (*n* = 4 mice), *p* = 0.1125; FRAX486‐WT (*n* = 3 mice) vs. FRAX486‐cKO (*n* = 3 mice), *p* = 0.8590; Layer V, Vehicle‐WT vs. Vehicle‐cKO, *p = 0.0141; FRAX486‐WT vs. FRAX486‐cKO, *p* = 0.7479; Layer VI, Vehicle‐WT vs. Vehicle‐cKO, *p* = 0.1040; FRAX486‐WT vs. FRAX486‐cKO, *p* = 0.7778. (d) Immunofluorescence for DAPI (blue), CUX1 (violet), CTIP2 (red), and TBR1 (green) in the M1 of WT and cKO mice at P6. Layers are identified by DAPI‐stained nuclear distribution pattern. Scale bar, 100 µm. (e) Thickness of different layers with FRAX486 or vehicle treatment. Unpaired *t*‐tests, CUX1, Vehicle‐WT (*n* = 3 mice) vs. Vehicle‐cKO (*n* = 4 mice), *p* = 0.5136; FRAX486‐WT (*n* = 3 mice) vs. FRAX486‐cKO (*n* = 4 mice), *p* = 0.9329; CTIP2, Vehicle‐WT vs. Vehicle‐cKO, *p* = 0.8255; FRAX486‐WT vs. FRAX486‐cKO, *p* = 0.7141; TBR1, Vehicle‐WT vs. Vehicle‐cKO, *p* = 0.2118; FRAX486‐WT vs. FRAX486‐cKO, *p* = 0.4644. (f) Quantification of layer‐specific neuronal density (#/mm^2)^ in the M1 of different groups at P6. Unpaired *t*‐tests, CUX1, Vehicle‐WT (*n* = 3 mice) vs. Vehicle‐cKO (*n* = 4 mice), *p* = 0.6788; FRAX486‐WT (*n* = 3 mice) vs. FRAX486‐cKO (*n* = 3 mice), *p* = 0.3928; CTIP2, Vehicle‐WT vs. Vehicle‐cKO, *p = 0.0305; FRAX486‐WT vs. FRAX486‐cKO, *p* = 0.3957; TBR1, Vehicle‐WT vs. Vehicle‐cKO, ***p = 0.0003; FRAX486‐WT vs. FRAX486‐cKO, *p* = 0.5207. n.s., no significant difference. All data are shown as mean ± SEM.

### 2.5 Impaired Motor Function in Adult *Arhgef1* cKO Mice

We next examined whether *Arhgef1* deletion from early corticogenesis leads to neurobehavioral changes in adulthood. 3‐month‐old WT and cKO mice were evaluated with a battery of behavioral tests including social behaviors, learning and memory, motor abilities, and mood status. The spontaneous exploratory locomotion of cKO mice was normal in the open‐field test (Figure 6a,b). cKO mice also exhibited normal social approach and social novelty in the Three‐chamber test, and cKO pups emitted normal ultrasonic vocalizations (USVs) over the first two postnatal weeks, suggesting that forebrain deletion of *Arhgef1* did not affect sociability and social communication (Figure S5a–f). Additionally, cKO mice showed no restricted repetitive behaviors, indicated by normal grooming and marble‐burying, suggesting a lack of autism‐like characteristics (Figure S5g,h). Regarding cognitive functions, the cKO mice exhibited normal object memory in the novel object recognition test, and normal spatial working memory in the Y‐maze alternation test (Figure S5i–m). These results suggest that forebrain *Arhgef1* is not important for social and cognitive functions.

**FIGURE 6:**
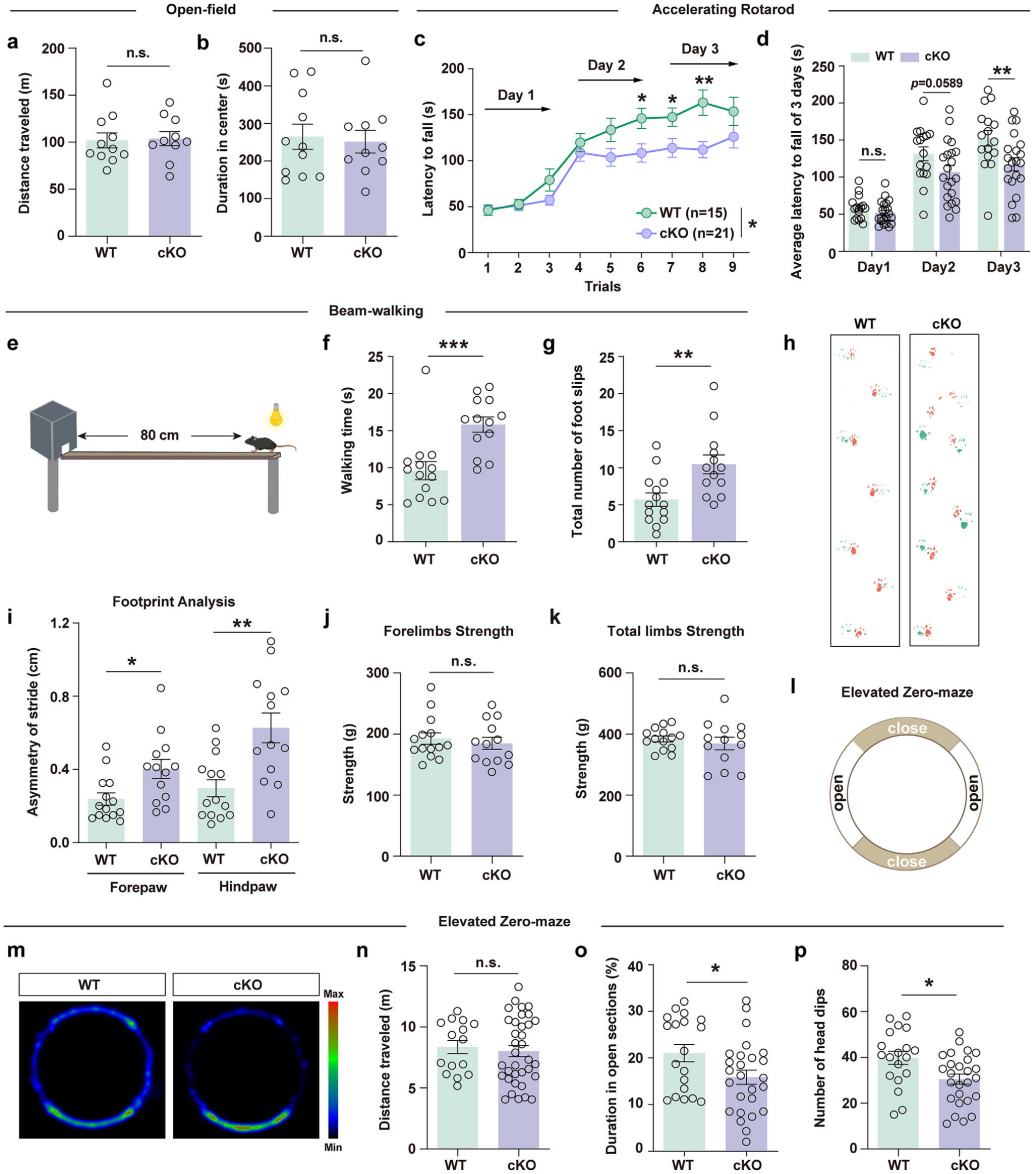
Impaired motor function and elevated anxiety in adult *Arhgef1* cKO mice. (a, b) Travel distance (a) and duration in the center region (b) of WT and cKO mice in the open‐field test. WT, *n* = 11 mice; cKO, *n* = 10 mice. Unpaired *t*‐tests, *p* = 0.8677 (a); *p* = 0.7755 (b). (c) Latency to fall in each trial across 3 test days of WT and cKO mice in the accelerating rotarod test. Two‐way ANOVA with Bonferroni's multiple comparison post hoc, *p = 0.0151. Unpaired *t*‐tests, trial 6, *p = 0.0171; trial 7, *p = 0.0284; trial 8, **p = 0.0025. (d) Average latency to fall on every test day of WT and cKO mice in the accelerating rotarod test. Unpaired *t*‐tests, day 1, *p* = 0.1704; day 2, *p* = 0.0589; day 3, **p = 0.0014. WT, *n* = 15 mice; cKO, *n* = 21 mice (c, d). (e) A cartoon illustration of the beam‐walking test. (f) Time for WT and cKO mice walking through the beam. Unpaired *t*‐test, ***p = 0.0007. (g) Number of foot slips made by WT and cKO mice during the beam‐walking test. Unpaired *t*‐test, **p = 0.0049. WT, *n* = 14 mice; cKO, *n* = 13 mice (f, g). (h) Representative footprints of WT and cKO mice. (i) Forepaw (red) and hindpaw (green) asymmetry of stride for WT and cKO mice was measured by the absolute difference between the stride lengths of left and right paws. WT, *n* = 14 mice; cKO, *n* = 13 mice. Unpaired *t*‐tests, forepaw, *p = 0.0133; hindpaw, **p = 0.0014. (j, k) Forelimbs (j) and total limbs (k) strength of WT and cKO mice in grip‐strength test. WT, *n* = 14 mice; cKO, *n* = 13 mice. Unpaired *t*‐tests, forelimbs strength, *p* = 0.5656; total limbs strength, *p* = 0.4970. (l) Schematic of the elevated Zero‐maze. (m) Representative heatmaps of WT and cKO mice activities. (n) Travel Distance of WT and cKO mice in the elevated Zero‐maze. Unpaired *t*‐test, *p* = 0.6700. (o) Duration spent in open sections by WT and cKO mice. Unpaired *t*‐test, *p = 0.0346. (p) Number of head dips made by WT and cKO mice in open sections. Unpaired *t*‐test, *p = 0.0110. WT, *n* = 15 mice; cKO, *n* = 35 mice. n.s., no significant difference. All data are shown as mean ± SEM.

Intriguingly, adult cKO mice presented prominent motor abnormalities in a series of motor function tests. In the accelerating rotarod test, whereas WT mice continued to stay longer on the rotating rods along the three testing days, cKO mice fell faster on both day 2 and day 3 (Figure 6c,d), suggesting compromised motor learning ability. In the beam‐walking test, cKO mice spent longer time walking through a long, narrow beam with more foot slips (Figure 6e–g). In the footprint analysis, although most parameters were normal, both the forepaw and hindpaw strides of cKO mice presented more severe asymmetry than WT mice, indicated by the larger absolute difference between the stride lengths of left and right paws (Figures 6h,i and S5n–p). All these results suggest that deletion of *Arhgef1* in the forebrain since early development led to deficits in motor learning, motor coordination and balance, and gait during adulthood. We further conducted a grip strength test and showed that the strength of both forelimbs or total limbs of cKO mice was similar to that of WT mice (Figure 6j,k), verifying that the motor deficits are originated in the forebrain but not resulted by weakened muscle strength. Besides the above behavioral analyses, we also measured the anxiety levels of WT and cKO mice. The cKO mice spent less time traveling in the open sections of the elevated Zero‐maze (Figure 6l–o), meanwhile, the exploratory moves characterized by the head dip, an action of leaning head out of the open corridors, were fewer than in WT mice (Figure 6p), suggesting higher anxiety levels in the cKO mice.

To further explore whether the behavioral abnormalities observed in adult cKO mice continue to be present during aging, 15‐month‐old WT and cKO mice were studied. Aged cKO mice still showed normal spontaneous exploration in the Open‐field test and normal cognitive function in the Y‐maze test (Figure S6a–d). In contrast to the increased anxiety in adulthood, the aged cKO mice performed similarly in the elevated Zero‐maze as WT mice, showing no change in anxiety levels (Figure S6e,f). Nonetheless, the aged cKO mice continuously displayed impaired motor‐learning ability in the accelerating rotarod test, falling faster on day 2 and day 3 compared to the WT mice (Figure S6g). Taken together, the absence of worsening of any behavioral deficits in aged mice implicates that ARHGEF1 may not be involved in aging or neurodegeneration processes, but the persistent motor dysfunction from adulthood to aged stages suggests that ARHGEF1 in early cortical development is essentially required for developing accurate motor functions in mice.

### 2.6 Altered Neuronal Morphology in the M1 of Adult *Arhgef1* cKO Mice

Given that deficits of motor function and reduced M1 deep‐layer neurons are prominent in *Arhgef1* cKO mice, we further examined morphological and functional changes of M1 neurons in adult cKO mice. We first analyzed dendrites complexity and dendritic spine density of Layer V pyramidal neurons (PNs) in the M1 of WT and cKO mice using Golgi staining (Figure 7a). Sholl analysis showed more complex dendrites of PNs in the M1 of cKO mice, with more branches and longer dendrites, compared with those of WT mice (Figure 7b–f). Intriguingly, although the total spine density increased markedly in PNs of cKO mice (Figure 7g,h), the density of mature (mushroom‐shaped) spines decreased while those of immature (thin and filopodial) spines increased significantly (Figure 7i). These results suggest an overgrowth of dendrites and spines with immature morphology in the M1 of cKO mice. Moreover, transmission electron microscope was used to access synaptic ultrastructure in the M1 of cKO mice and WT mice. The number of postsynaptic density (PSD) of asymmetric synapses, which normally represent excitatory synapses, was reduced in the M1 of cKO mice, accompanied by thinner PSD thickness and unaltered PSD length (Figure 7j–m). The decrease in PSD thickness and length of symmetric synapses also suggests a probable impairment of inhibitory synapses in the cKO mice, although the PSD number was normal (Figure 7j,k,n,o). These results collectively revealed morphological abnormalities of dendrites, dendritic spines, and postsynaptic organization of PNs in the M1 of *Arhgef1* cKO mice.

**FIGURE 7:**
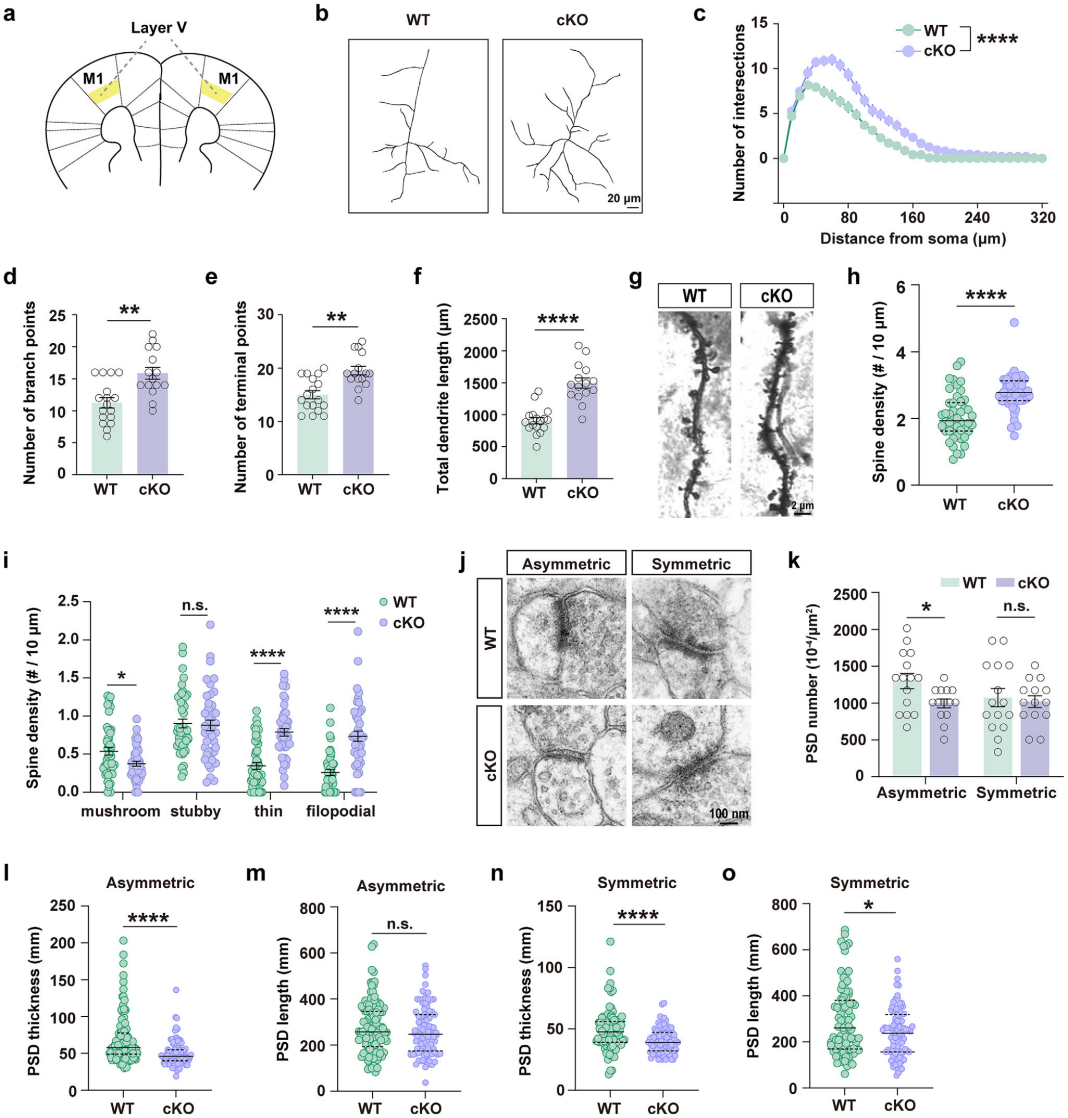
Altered neuronal morphology in the M1 of adult *Arhgef1* cKO mice. (a) Pyramidal neurons from Layer V of the M1 of adult WT and cKO mice were analyzed. (b) Representative images of reconstructed Golgi‐stained pyramidal neurons. Scale bar, 20 µm. (c) Number of dendritic intersections in the M1 of WT and cKO mice obtained by Sholl analysis. WT, *n* = 17 neurons from 3 mice; cKO, *n* = 15 neurons from 3 mice. Two‐way ANOVA with Bonferroni's multiple comparison test, ****p < 0.0001. (d–f) Number of dendritic branch points (d), terminal points (e), and total dendrite length (f) of neurons in the M1 of WT and cKO mice. Mann–Whitney tests, number of branch points, **p = 0.0016; number of terminal points, **p = 0.0011. Unpaired *t*‐tests, dendrite length, ****p < 0.0001. (g) Representative images of Golgi‐stained dendrite spines of WT and cKO mice neurons. Scale bar, 2 µm. (h) Total spine density (#/10 µm) of WT and cKO neurons. WT, *n* = 46 dendrites from 3 mice; cKO, *n* = 44 dendrites from 3 mice. Mann–Whitney test, ****p < 0.0001. (i) Spine density of different morphological subtypes (mushroom, stubby, thin, filopodial). Mann–Whitney tests, mushroom, *p = 0.0363; stubby, *p* = 0.7207; thin, ****p < 0.0001; filopodial, ****p < 0.0001. (j) Electron microscopic images of asymmetric and symmetric synapses in the M1 of adult WT and cKO mice. Scale bar, 100 nm. (k) Number of asymmetric and symmetric postsynaptic density (PSD). WT, *n* = 15 views from 3 mice; cKO, *n* = 14 views from 3 mice. Mann–Whitney tests, asymmetric, *p = 0.0375; symmetric, *p* = 0.9566. (l, m) Quantification of asymmetric PSD thickness (l) and length (m). WT, *n* = 116 PSD from 3 mice; cKO, *n* = 95 PSD from 3 mice. Mann–Whitney test, thickness, ****p < 0.0001. Unpaired *t*‐test, length, *p* = 0.4228. (n, o) Quantification of symmetric PSD thickness (n) and length (o). WT, *n* = 92 PSD from 3 mice; cKO, *n* = 95 PSD from 3 mice. Mann–Whitney test, thickness, ****p < 0.0001. Unpaired *t*‐test, length, *p = 0.0421. n.s., no significant difference. All data are shown as mean ± SEM.

### 2.7 Abnormal Neuronal Activities in the M1 of Adult *Arhgef1* cKO Mice

Followed by the morphological analyses, we recorded neuronal activities in the M1 of cKO mice. First, we used in vivo two‐photon calcium imaging to record neuronal calcium signals of PNs in the M1. Excitatory neurons in the M1 of both WT and cKO mice were expressed with the calcium sensor GCaMP6s under the CaMKIIα promoter (rAAV2/9‐CaMKIIa‐GCaMP6s), and the dynamic changes of calcium signals in layer II/III and layer V PNs of M1 were recorded and analyzed in awake, head‐fixed mice (Figure 8a,b). Interestingly, the spontaneous calcium activities and peak values were weakened in Layer II/III PNs but enhanced in layer V PNs of M1 in cKO mice when compared to WT mice. The frequency of calcium spikes of both Layer II/III and Layer V PNs was not altered in cKO mice (Figure 8c–h). These neuronal activity changes at resting state were consistent with the morphological results that layer V PNs have more complex dendrites and total spines in the M1 of cKO mice.

**FIGURE 8:**
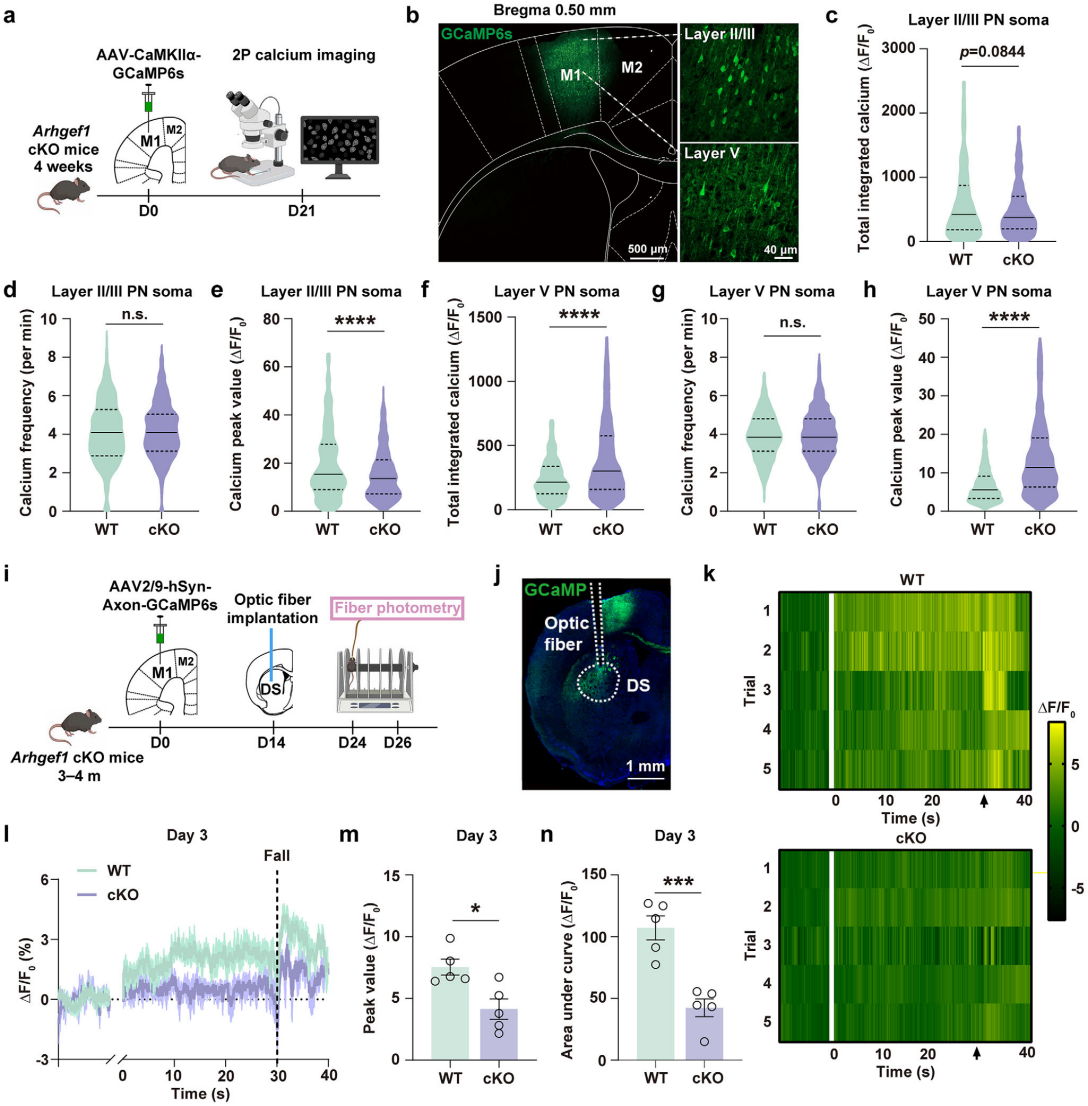
Abnormal neuronal activities in the M1 of adult *Arhgef1* cKO mice. (a) Schematic of in vivo two‐photon calcium imaging in the M1. (b) A representative brain section showing prominent expression of GCaMP6s in the M1. Scale bar, 500 µm in low‐magnification views, 40 µm in enlarged views. (c) Total calcium activities (in ΔF/F_0_) of pyramidal neuron somas from layer II/III in the M1 of WT and cKO mice. Mann–Whitney test, *p* = 0.0844. (d) Calcium spike frequency (total spike number per min) of layer II/III neurons. Mann–Whitney test, *p* = 0.2923. (e) Peak value of calcium spikes of layer II/III neurons. Mann–Whitney test, ****p < 0.0001. WT, *n* = 671 neurons from 5 mice, cKO, *n* = 680 neurons from 7 mice (c–e). (f) Calcium activities of layer V pyramidal neurons in the M1 of WT and cKO mice. Mann–Whitney test, ****p < 0.0001. (g) Calcium spike frequency of layer V neurons. Mann–Whitney test, *p* = 0.2071. (h) Peak value of calcium spikes of layer V neurons. Mann–Whitney test, ****p < 0.0001. WT, *n* = 671 neurons from 5 mice, cKO, *n* = 680 neurons from 7 mice (f–h). (i) Schematic of corticostriatal calcium signal recording during the accelerating rotarod test. (j) A representative brain section showing expression of GCaMP6s in the cell soma of M1 and projection fibers in the dorsal striatum (DS). Scale bar, 1 mm. (k) Representative heatmaps of normalized calcium activities (ΔF/F_0_) from WT and cKO mice before and after falling (indicated by an arrow). Data from five mice are shown, each aligned to the time of falling from the rod (arrows). (l) Averaged ΔF/F_0_ from WT and cKO mice. (m) Peak value (ΔF/F_0_) of corticostriatal calcium signals from WT and cKO mice. Unpaired *t*‐test, *p = 0.0122. (n) Total calcium activities of the corticostriatal pathway measured by the area under the curve (ΔF/F_0_) from WT and cKO mice. *n* = 5 mice per group. Unpaired *t*‐test, ***p = 0.0007. n.s., no significant difference. All data are shown as mean ± SEM.

Next, we asked how neuronal activities induced by motor tasks are altered in the cKO mice. The execution of voluntary movement depends on the classic M1‐dorsal striatum (DS) circuit in which axons of M1 PNs project to activate DS neurons. Since activities of this circuit were dynamically modulated during motor‐learning progress, we recorded in *vivo* calcium activities using fiber photometry in the accelerating rotarod test. An axon‐enhanced GCaMP6s virus (rAAV‐hSyn‐axon‐GCaMP6s) was expressed in the DS‐projecting axon tracts of M1 neurons, and the optic fibers were implanted into DS for corticostriatal calcium signal recording during the accelerating rotarod test (Figure 8i,j) [35]. It was observed that activities of M1 PN terminals in the DS reached a maximal peak when mice fell from the rotating rods. Hence, we analyzed calcium signals in the period spanning the 30 s before and 10 s after the fall, and assigned these signals as motor‐related activities (Figure 8k,l). Calcium signals in the 10 s before the accelerating phase were quantified as baseline. Notably, the motor‐related corticostriatal calcium activities, both the peak and total values (area under the curve), were remarkably decreased in the cKO mice on test day 3 (Figure 8m,n). These findings demonstrate a crucial role of ARHGEF1 in the formation of corticostriatal circuitry for motor learning.

## 3 Discussion

Abnormal motor development is highly represented in ASD, ADHD, and other NDDs. However, the developmental mechanisms of motor function have been poorly studied. In the present study, we identify a critical developmental period during which ARHGEF1 is indispensable for deep‐layer cortical neurogenesis via restricting RAC1/CDC42‐PAK1 signaling. We further demonstrate that ARHGEF1 is important for proper morphogenesis and functional activities of motor cortical neurons, thereby modulating motor learning and coordination. These findings collectively unravel ARHGEF1 as a key determinant of early cortical neurogenesis, motor circuit formation, and motor function.

The major cortical developmental defects of *Arhgef1* cKO mice are decreased neurogenesis, accelerated cell cycle exit, and premature differentiation of deep‐layer cortical neurons. While the cell number decreases, the dendrites complexity and the total spine density increase in Layer V neurons of M1 of cKO mice in adulthood, together with elevated spontaneous calcium activity at rest. Consistently, our previous studies have shown that *Arhgef1* knockdown induces neural differentiation and neurite outgrowth [31, 32]. The overgrowth of neurites/dendritic branches and spines, together with altered basal neuronal activity, could originate from early deletion of *Arhgef1* and the hyperactivation of the PAK1‐cofilin pathway, or it could be from late‐onset defects induced by *Arhgef1* deficiency. It is important to note that the increase in spine density was predominantly attributable to immature spines, whereas the mature spine number and PSD content were reduced, suggesting impairment of maturation and synaptic transmission of M1 Layer V neurons despite the overall increase in dendritic protrusions. Accordingly, as Layer V PN neurons are the main output neurons of the corticostriatal circuit for motor control, the abnormal basal activity and decreased functional synapses of these neurons suggest that they may not respond efficiently to motor stimulation. Indeed, fiber photometry recordings showed decreased movement‐evoked calcium activities in M1‐DS projection fibers of cKO mice during rotarod running. The increased spontaneous activity at rest and reduced movement‐evoked activity may reflect distinct aspects of neuronal dysfunction rather than opposing physiological outcomes. Resting somatic calcium activity reflects the basal excitability and spontaneous ongoing network state of Layer V neurons, whereas movement‐evoked calcium signals recorded from M1–DS terminals reflect behavior‐dependent recruitment of the cortical‐striatal circuit. In fact, spontaneous neural activity does not necessarily correlate with behavior‐associated responses. Similar dissociations have been reported in models of neurodevelopmental dysfunction. In *Cntnap2* ^−/−^ mice, spontaneous c‐Fos expression is elevated in the somatosensory cortex, whereas whisker stimulation fails to elicit a corresponding increase in c‐Fos activation or calcium responses [36]. Likewise, elevated spontaneous activity but reduced reliability of sensory responses is observed in *Fmr1^−/y^* mice [37]. These findings suggest that elevated basal activity can coexist with impaired processing of behavior‐related information. In contrast to the hyperexcitation of layer V neurons, the layer II/III neurons have slightly decreased spontaneous calcium activity, suggesting an imbalanced interlaminar activity change and defective interconnectivity between II/III and V layers in *Arhgef1* cKO mice. Therefore, Emx1‐Cre‐driven forebrain deletion of *Arhgef1* leads to impaired neurogenesis, morphology, and function of Layer V PNs in M1, which may contribute to compromised circuit activation in response to motor stimulation and culminate in motor deficits.

Although it remains to be determined whether the adult neuronal abnormalities and motor deficits arise from early developmental defects, our findings suggest that early developmental abnormalities likely contribute substantially to the neuronal dysfunctions in adult *Arhgef1* cKO mice, because (1) ARHGEF1 is most highly expressed during early cortical development, and the **alteration** of RHOA/RAC1/CDC42 and PAK1‐Cofilin signaling is also most pronounced in early cortical development following deletion of *Arhgef1*; (2) only early‐born deep‐layer but not late‐born upper‐layer neurons are affected in the cKO mice; (3) *Arhgef1* cKO mice exhibit no progressive motor deterioration as aging. However, we could not exclude the possibility that ARHGEF1 may also participate directly in the regulation of synapse morphogenesis and function at later developmental stages. Synaptic localization of ARHGEF1 has been reported in human brain organoids [38], but its function on synapses has not been explored. Further studies using approaches to manipulate ARHGEF1 in later stages will be required to delineate how it regulates motor development from early embryonic to adult stages.

RAC1/CDC42‐PAK1 axis is one of the core pathways regulating neuronal development by transducing neural signals to the cytoskeleton. This pathway is closely related to NDDs, playing important roles in neuronal morphogenesis, migration, and synapse formation [39]. Although bidirectional alterations of the RAC1/CDC42‐PAK1 axis perturb corticogenesis and neuronal development, the temporal controls of this signaling during different developmental windows have not been fully elucidated. In the current study, we revealed that ARHGEF1 is a specific GEF particularly important for the negative regulation of cortical RAC1/CDC42‐PAK1 activity during embryonic stages, and this effect is sharply decreased at neonatal stages and disappears in adulthood. This regulatory pattern coincides with the developmental expression profile of ARHGEF1, which is high during early corticogenesis and gradually decreases afterward. Consistently, RHOA, the direct target of ARHGEF1, is strongly inhibited at the embryonic stage but not at P6 and in adulthood. It has been demonstrated that RHOA and RAC1 mutually inhibit each other [16, 17, 18]. Because in vitro small GTPase assays have confirmed that ARHGEF1 does not directly modulate RAC1 and CDC42 activities [34], the robust activation of RAC1 and CDC42 at early corticogenesis may be resulted by the RHOA inhibition. However, it is interesting to note that RAC1 and CDC42 were still upregulated in cKO mice at P6 when RHOA no longer showed a change. This could be due to a prolonged disinhibition of RAC1 by the previous strong RHOA suppression. Furthermore, it has been reported that active RAC1 promotes the binding of PAK1 to ARHGEF1, thus inhibiting the ability of ARHGEF1 to activate RHOA [40]. Hence, there is a possibility that the active RAC1 at P6 does not result in RHOA inhibition due to the absence of ARHGEF1. Alternatively, the normalization of RHOA/RAC1/CDC42 activity at later developmental stages may result from late‐onset expression of compensatory RHOA GEFs or RAC1/CDC42 GAPs.

Although members of Rho GEFs and GAPs are critical regulators of many neurobehaviors, their roles in motor functions are poorly studied. Only limited Rho family regulators are known to participate in motor control. For example, persistent RAC1 activation is observed in Rich2 (a RAC1 GAP) knockout mice, and these mice show impaired motor learning ability [41]. In addition, Vav3 and P‐Rex2 (both RAC1 GEFs) knockout mice also show decreased motor ability and/or gait abnormalities [42, 43]. In this study, we revealed ARHGEF1 as a new regulator of the Rho family in motor development. Forebrain deletion of *Arhgef1* specifically affects motor functions and anxiety in adulthood, whereas social interaction, language communication, and cognitive functions are normal. Interestingly, motor deficits are persistently present during aging, whereas anxiety is recovered to normal levels in 15‐month‐old mice. These findings suggest that ARHGEF1 is specifically crucial for lifelong motor functions, offering new knowledge of Rho family regulation in motor control. It is noteworthy that both MalaCards and GeneCards databases recognize *ARHGEF1* as a potential gene linked to Giant Axonal Neuropathy (GAN), an infantile to early‐childhood‐onset motor condition with symptoms of impaired mobility and reduced motor coordination [44]. Our *Arhgef1* cKO mice indeed display some of the characteristics of motor deficits in GAN, such as impaired motor learning and coordination. Nonetheless, there is so far no direct clinical evidence or gene variants of *ARHGEF1* identified in GAN patients, and its linkage to GAN is inferred based on an interactome study of Gigaxonin, encoded by the disease‐causing *GAN* gene [44]. To understand whether ARHGEF1 contributes to the pathogenesis of GAN, its interaction with Gigaxonin needs to be first verified. It is then essential to determine whether this interaction is important for Gigaxonin function on the regulation axon morphology, and whether disruption of this interaction leads to hallmark GAN‐related axonal abnormalities.

PAK1 inhibitors have shown promising therapeutic effects in various neurological diseases. Fibroblasts derived from two NDD patients with *PAK1* mutations exhibit enrichment of filopodia, a cytoskeletal characteristic mainly caused by the activation of the RAC1‐PAK1 pathway, whereas the PAK1 inhibitor FRAX486 completely reverses this phenotype [22]. FRAX486 also restores dendritic spine morphology and alleviates behavioral abnormalities such as epilepsy, hyperactivity, stereotyped movements, and somatosensory dysfunctions in *Fmr1* KO mice, a model of fragile X syndrome [27, 28]. Other PAK1 inhibitors are also reported to improve dendritic spine abnormalities and prepulse inhibition behavior in a schizophrenia mouse model [25]. Additionally, inhibition or knockout of PAK1 has been found to improve social function in a neurofibromatosis type 1 mouse model and shows potential applications in neurodegenerative diseases and pain [26, 45, 46]. In the current study, we revealed that application of FRAX486 during early corticogenesis corrects abnormal early‐born neurogenesis and subsequently restores deep‐layer cortical neuron numbers in the M1 of *Arhgef1* cKO mice. These findings identify that PAK1‐dependent signaling must be properly controlled to ensure early cortical development, and suggest PAK1 as a potential target for correcting developmental abnormalities caused by *Arhgef1* deficiency. However, it remains to be determined whether early PAK1 inhibition is sufficient to ameliorate the neuronal morphological and activity abnormalities, as well as the motor circuit and behavioral deficits in the cKO mice in adulthood. Further studies assessing these long‐term functional outcomes after FRAX486 treatment in specific developmental phases will be required to establish whether correction of early cortical abnormalities translates into sustained improvements in adult neural and motor function. Taken together, by pinpointing a critical developmental window in which ARHGEF1 acts on deep‐layer cortical neurogenesis through restricting PAK1 activity, this study offers new insight into the molecular regulation of corticogenesis and motor development.

## 4 Materials and Methods

### 4.1 Generation of *Arhgef1* cKO Mice

*Arhgef1* cKO (*Arhgef1* ^fl/fl^; *Emx1‐Cre* ^+/−^) mice and their WT (*Arhgef1* ^fl/fl^; *Emx1‐Cre* ^−/−^) littermates were generated using Cre‐LoxP strategy through crossbreeding of *Arhgef1*‐floxed (*Arhgef1* ^fl/fl^) mice and *Emx1‐Cre* ^+/−^ mice. The *Arhgef1*‐floxed mice were bred from *Arhgef1^fl/fl^; LysM‐Cre* mice [47], generously provided by Prof. Raul M. Torres from University of Colorado Health Sciences Center, and the *Emx1‐Cre* mice by Prof. Yibo Qu and Prof. Libing Zhou from Guangdong‐Hong Kong‐Macau Institute of CNS Regeneration, Jinan University. All animal experiments were conducted in accordance with the Laboratory Animal Ethics Committee of Jinan University.

### 4.2 RNA Extraction and Quantitative Real‐Time PCR (qPCR)

Cortical, hippocampal, and striatal tissues were quickly dissected out and homogenized in RNAiso PLUS (Takara, 9108). cDNA was synthesized using M‐MLV Reverse Transcriptase Kit (Promega, M1701). qPCR was carried out using SYBR Green qPCR Master Mix (Bimake, B21202) following the manufacturer's instructions. The amplification reaction was quantified by a Light Cycler 480 Real‐Time PCR System (Roche), with *Gapdh* serving as an internal control. The primer *Arhgef1*‐RT targeted Exon4‐Exon5 of all *Arhgef1* isoforms. The primer sequences used for qPCR are as follows (from 5’ to 3’):

*Arhgef1*‐RT‐F: GCCTAGAGCAAGTGAAGCG
*Arhgef1*‐RT‐R: TAGAACCGCAGTCTTCTCCAG
*Gapdh*‐F: GAGCCAAAAGGGTCATCATCTC
*Gapdh*‐R: GTGAGCTTCCCGTTCAGCTCT

### 4.3 Western Blotting

Protein extraction and Western blotting procedures were performed as previously described [48]. All primary antibodies used in Western blotting are as follows: ARHGEF1 (3669S, 1:1000 dilution), CDC42 (2466S, 1:1000 dilution), Cofilin (5175S, 1:1000 dilution), PAK1 (2602S, 1:1000 dilution), p‐Cofilin (Ser3, 3313S, 1:1000 dilution), and RHOA (2117S, 1:1000 dilution) were from Cell Signaling Technology (Danvers, MA, USA); β‐actin (A01010, 1:5000 dilution) was from Abbkine (Wuhan, China); p‐PAK1 (Ser199, ab192814, 1:1000 dilution) was from Abcam (Cambridge, UK). RAC1 (610650, 1:1000 dilution) was from BD Biosciences (San Jose, CA, USA). The secondary antibodies, including anti‐mouse IgG HRP (7076S, 1:2000 dilution) and anti‐rabbit IgG HRP (7074S, 1:2000 dilution), were from Cell Signaling Technology.

### 4.4 Immunofluorescence

Immunofluorescence and colocalization analysis were conducted as described previously [49]. The primary antibodies used were as follows: ARHGEF1 (ab243248, 1:100 dilution), CUX1 (11733‐1‐AP, 1:500 dilution), CTIP2 (ab18465, 1:200 dilution), TBR1 (ab31940, 1:500 dilution), GAD65/67 (ab183999, 1:500 dilution), Ki67 (ab16667, 1:200 dilution), p‐PAK1 (Ser199, ab192814, 1:100 dilution), and SOX2 (ab97959, 1:200 dilution) were from Abcam; IBA1 (MABN92, 1:200 dilution), NeuN (MAB377, 1:500 dilution), and OLIG2 (AB9610, 1:200 dilution) were from Merck Millipore (Billerica, MA, USA); CaMKIIα (50049S, 1:200 dilution), p‐Cofilin (Ser3, 3313S, 1:100 dilution), and pH3 (3377S, 1:200 dilution) were from Cell Signaling Technology; GFAP (GB12096, 1:500 dilution) was from Servicebio (Wuhan, China). Rhodamine‐Phalloidin (R415), DAPI (D9542) and the secondary antibodies including Alexa Fluor 546 Goat Anti‐Mouse IgG (A11030, 1:500 dilution), Alexa Fluor 488 Goat Anti‐Rabbit IgG (A11034, 1:500 dilution), and Alexa Fluor 647 Goat Anti‐Rabbit IgG (A21245, 1:500 dilution) were from Invitrogen (Waltham, MA, USA).

### 4.5 Neurogenesis and Neuronal Migration Analysis

Adult female *Arhgef1* cKO mice and male WT were crossbred to obtain fetal WT and cKO littermates. Pregnant mice were intraperitoneally (i.p.) injected with EdU (50 mg/kg in saline, Beyotime, ST067) for birthdating analysis. To evaluate S‐phase proliferative cells, EdU was injected at E13.5 or E16.5, and the embryos were collected 1 h later to prepare cryostat brain sections for EdU labeling with Alexa Fluo azide 647 by click reaction (Thermo, C10340). To evaluate cell cycle exit, EdU was injected at E12.5 or E16.5, and the embryos were analyzed 24 h later for EdU labeling and immunostaining of Ki67, a marker of cells within cell cycles. The cell fractions that have quit the cell cycle were calculated by EdU^+^Ki67^−^ cells/total EdU^+^ cells. To evaluate neuronal migration, EdU was injected at E12.5 or E16.5, and the offspring were sacrificed at P6 for EdU labeling. Additionally, pH3 staining was performed at either E13.5 or E16.5 to measure M‐phase proliferative cells.

### 4.6 RHOA/RAC1/CDC42 Activity Assay

Cortical tissues from E14.5, P6, and adult (3–4 months old) mice, and hippocampal tissues from P6 and adult mice were subjected to the RHOA/RAC1 assay following our published protocols [48, 50, 51]. GST‐tag Purification Resin (BeyoGold, P2253) coupled with the Rho‐binding domain of Rhotekin (GST‐RBD) and the p21 binding domain of PAK1 (GST‐PBD) were used to specifically pulldown active RHOA (GTP‐RHOA) and RAC1/CDC42 (GTP‐RAC1/CDC42), respectively. Levels of GTP‐bound RHOA, RAC1, and CDC42 were normalized to their respective total protein levels to determine activity of each.

### 4.7 PAK1 Inhibition

Adult female *Arhgef1* cKO mice and male WT were crossbred to obtain WT and cKO fetal littermates. FRAX486 (MCE, HY‐15542B) was dissolved in vehicle (20% hydroxypropyl‐β‐cyclodextrin (Macklin, 128446‐35‐5) and 5% DMSO (Sigma, D4540) in normal saline) to a concentration of 1 mg/mL [25, 27, 28]. To examine the rescue effect of PAK1 inhibition on cell proliferation in cKO embryos, pregnant mice were i.p. injected with FRAX486 (10 mg/kg, i.e., 10 µL/g body weight) or an equal volume of vehicle (10 µL/g body weight) at gestational day 12.5. For analysis of proliferative cell number, EdU (50 mg/kg) was i.p. administered at 24 h after FRAX486 injection, and the embryonic brains were collected at 1 h later for EdU labeling. For cell cycle quit fraction assessment, EdU was administered at 2 h after FRAX486 injection, and the embryonic brains were collected 24 h later for EdU labeling and Ki67 immunostaining later. To examine the rescue effect of PAK1 inhibition on cortical lamination and neuronal migration, FRAX486 (10 mg/kg) or vehicle was i.p. administered to the pregnant mice at gestational days 12.5, 14.5, and 17.5 (10 mg/kg, once each day), and EdU was injected 2 h after the first FRAX486 injection. The offspring were sacrificed at P6 for EdU labeling and analysis.

To validate the success of in utero inhibition of PAK1 phosphorylation by FRAX486, we i.p. injected the drug to the pregnant mice at E12.5 and collected the embryonic cortical tissues at E14.5. P‐PAK1 and p‐Cofilin were examined by Western blotting.

### 4.8 Behavior Tests

Adult (3–5 months old) WT and cKO littermates were housed individually for 3 days and handled for 5 min each day before behavior tests, measuring different functions in the following order: spontaneous and social behaviors, learning and memory, motor function, and anxiety [48, 49, 52]. USVs of pups induced by maternal separation were recorded during the first two postnatal weeks [53]. Before the start of each testing day, mice were habituated to the test room for at least 30 min. Experimenters were blind to genotypes.

### 4.9 Open‐Field Test

Mice were placed in a white box (40 cm × 40 cm × 40 cm) and allowed to explore the arena for 30 min as previously described [48, 49, 52]. The time spent in the center and the total distance of movement were evaluated by Topscan 3.0 (Clever Sys Inc).

### 4.10 Accelerating Rotarod Test

The ability of motor learning was evaluated by the accelerating rotarod test. The mouse was first placed on a rotating rod (Ugo Basile, 47650) at a low speed of 4 rpm for 1 min to adapt. To commence the test, the rotating rod was set to accelerate from 4 to 80 rpm in 5 min. The latency to fall was recorded as the time when the mouse fell from the rod or kept grasping the rod for two circles. The whole test lasted for three consecutive days, and three trials with 30 min inter‐trial intervals were taken on each testing day.

### 4.11 Beam‐Walking Test

Motor coordination and balance of mice were measured using horizontal beams of 100‐cm‐long and 16‐mm‐wide, suspended 50 cm above the ground. Mice were allowed to walk on the beam from one end to the other. Each mouse was tested three times a day, with 30 s inter‐trial intervals, for three consecutive days. Time of crossing 80 cm of the beam and the number of foot slips were calculated.

### 4.12 Footprint Analysis

The gait of mice was evaluated by footprints on a 42‐cm‐long, 4.5‐cm‐wide runway covered by paper. Before the test, mice were trained to walk through the runway once. Then the paws of mice were dipped with nontoxic acrylic resin (forepaws in red, and hindpaws in green) and allowed to walk through the runway to leave paw prints on the paper. Three trials were conducted for each mouse. At least 3–5 steps on each paper were analyzed to obtain the distance between two consecutive footprints of the same paw (stride length), the absolute difference between the stride lengths of left and right paws (asymmetry of stride length), the distance between the paired footprints of the two forepaws or two hindpaws (base width), and the distance between the paired footprints of the forepaws and hindpaws from the same body side (overlap).

### 4.13 Grip‐Strength Test

The grip strengths of forelimbs and total limbs of the mice were measured by a Grip Strength Meter (Jinan Yiyan Technology, YLS‐13A). For the test of forelimbs, the mouse was picked up by the tail and put on the wire of the Grip Strength Meter, with the hindlimbs remaining suspended. The mouse tail was slowly pulled back until the forelimbs left the wire, when the grip strength value was recorded. Each mouse underwent 5 repeated trials with 10‐second inter‐trial intervals between each trial, and the grip strength was calculated as the average value of all trials. For the test of total limbs, the procedures were the same except that all limbs were put on the wire to measure grip strength.

### 4.14 Elevated Zero‐Maze

This test was performed as previously described [48, 49, 52]. The maze is a 5.5‐cm‐wide circular corridor with a diameter of 53 cm, which is 60 cm high from the floor. The corridor is divided equally into two open sections and two closed sections arranged alternately. Each mouse was placed in one of the junctions of open and closed sections and allowed to explore for 5 min. The time and distance traveled by the mice in open and closed sections, as well as head dips in the open sections, were analyzed by Topscan 3.0.

### 4.15 Golgi‐Cox Staining and Analysis

In brief, mice brains were fixed and dyed for 14 days using a Golgi‐Cox staining kit (Servicebio, G1069) at room temperature. 60‐µm‐thickness brain slices were prepared using a vibrating microtome (Leica VT1200S), followed by soaking in the Golgi developer solution for 30 min and finally mounted with glycerin gelatin. The selected brain areas were imaged under bright‐field by Zeiss microscope system (Zeiss Axio Observer). For spine density assessment, images were captured with a 63× oil‐immersed objective and analyzed using Fiji 1.52p software (Rawak Software). For Sholl analysis of dendrite morphology, images were captured with 20× objective and assessed with Imaris 9.1 software (Bitplane).

### 4.16 Transmission Electron Microscopy

M1 were micro‐dissected out and cut into small blocks (less than 1 mm^3^), and then fixed in EM fixative solution (Servicebio, G1102) for 1.5 h at room temperature. Then tissue blocks were dehydrated in a graded ethanol series (30%–100%) and acetone, followed by penetration and embedding in EMBed‐812 (SPI‐Chem, 90529‐77‐4). The resin blocks were cut into ultrathin sections (60–80 nm) with an ultramicrotome (Leica EM UC7) and mounted onto cuprum grids. After being stained with 2% uranium acetate saturated alcohol solution and 2.6% lead citrate in sequence, selected brain areas were imaged under a transmission electron microscope (HITACHI HT7800). Synaptic morphological measurements were conducted using Fiji 1.52p software (Rawak Software).

### 4.17 In Vivo Two‐photon Calcium Imaging

1‐month‐old WT and cKO mice were anesthetized with an i.p. injection of 1.25% tribromoethanol and then head‐fixed in a stereotaxic apparatus (RWD Life Science). The M1 was located (AP: +1.5 mm, ML: +1.5 mm, DV: −0.55 mm) and 150 nL rAAV2/9‐CaMKIIa‐GCaMP6s (5.21 × 10^12^ vg/mL, BrainVTA) was injected through a glass micropipette connected to a microinjection pump at a rate of 40 nL/min. Then the micropipette remained in position for 10 min before being withdrawn. After 3 weeks, mice were anesthetized again, and the skull over the motor cortex was removed and replaced with a piece of glass coverslip (1.5 mm diameter). The next day, mice were put on the platform of the microscope for adaptation. 30 min later, a 20× water‐immersed objective of a two‐photon microscope (Zeiss LSM780) with 920‐nm‐length excitation wavelength and 2100‐mW‐power laser was used to record calcium activities activity of neuronal somas in the M1 of awake mice for 150 s. The recordings were analyzed using suite2p (version 0.10.1) for neuron extraction. Approximately 50–150 neurons per field‐of‐view were included in the analysis. Fluorescence signals from each identified neuron were corrected by subtracting 70% of the corresponding neuropil signal and were normalized to ΔF/F_0_ using a baseline defined as the mode of the calcium signal. The sum of ΔF/F_0_ values across the recording period for each neuron was calculated as the total integrated calcium signal. Neurons with negative total integrated calcium values were excluded from further analysis. Calcium peak amplitude was defined as the maximum value of the ΔF/F_0_ trace. For calcium event frequency analysis, events were defined as peaks exceeding three times the standard deviation of the ΔF/F_0_ signal, and event frequency was calculated by normalizing the total number of detected events to the imaging duration.

### 4.18 Fiber Photometry

1‐month‐old WT and cKO mice received stereotaxic injections of 200 nL rAAV‐hSyn‐axon‐GCaMP6s‐WPRE‐hGH pA (2.78 × 10^12^ vg/mL, BrainVTA) in the M1 (AP: +1.5 mm, ML: +1.5 mm, DV: −0.55 mm) at a rate of 25 nL/min. After 2 weeks, mice received a stereotaxic implant of an optic fiber (3 mm diameter) in the dorsal striatum (AP: +0.5 mm, ML: +2.0 mm, DV: −3.0 mm). 10 days later, calcium activities of M1 projection fibers in the dorsal striatum were recorded during the accelerating rotarod test. In brief, mice were put in the experiment room for 30‐min habituation. The acquisition aperture of the optic fiber was corrected in the dark, and the powers of the 410‐nm reference laser and the 470‐nm recording laser were adjusted to 10–20 µW and 20–40 µW, respectively. Then the optic fiber was connected to the instrument (Inper Signal) for recording while the mice were running on the rotarod apparatus, which was programmed to roll for 90 s at a low and steady speed (2 rpm) followed by accelerating to 80 rpm in 5 min. Each animal was tested 3 times with 30‐min inter‐trial intervals. The calcium signals were recorded and analyzed by Inper Plot v 1.0.1 (Inper Ltd.). The calcium activities from the 10‐s duration just before the accelerating phase were set as baseline (F_0_), and those from the duration including 30 s before and 10 s after falling were considered as motor‐related calcium signals (F). Peak value and area under the curve (AUC) were calculated as ΔF/F_0_. GraphPad Prism 9.0 (GraphPad Software) was used to create heat maps.

### 4.19 Statistical Analysis

The statistical analyses were performed using GraphPad Prism 9.0. All data were tested for Gaussian distribution, and *F* tests were used to compare variances. For normal distribution with similar standard deviation (SD), statistical comparisons for two groups or multiple groups were conducted using unpaired *t*‐test or one‐way analysis of variance (ANOVA), respectively. For normal distribution with significant various SD, Welch's *t*‐test or Welch's ANOVA test was conducted for two‐ or multiple‐group comparisons, respectively. For non‐normal distribution, data were analyzed using the Mann–Whitney test or Kruskal–Wallis test for two‐ or multiple‐ group comparison, respectively. For analysis involving two factors, two‐way ANOVA was applied with Bonferroni's multiple comparison. Statistical significance was considered when *P *< 0.05.

## Author Contributions

L. L. and X. M. contributed equally to this work. Conceptualization: L. L., X. M., and L. S. Project administration: L. S. Data curation/Investigation: L. L., X. M., Z. D., B. S., L. L., H.M. Y., T. W., Y. G. Formal analysis: L. L., X. M., H.M. Y. Resources: J.P.K. I., L. Z., Y. P., S. Z., K. L., L. S. Methodology: L. L., X. M., L. S. Supervision: J.P.K. I., L. Z., L. S. Writing: L. L., X. M., W. W., L. S. Funding acquisition: S. Z., X. M., Y. P., L. S.

## Funding

This work was supported in part by the Brain Science and Brain‐like Intelligence Technology‐National Science and Technology Major Project (2022ZD0214400), the National Key Research and Development Program of China (2022YFA1104900), the National Natural Science Foundation of China (82371175, 82401804, U24A20804), Guangdong Basic and Applied Basic Research Foundation (2022B1515130007, 2023B1515040015, 2023A1515111179, and 2026A1515010645), the Science and Technology Program of Guangzhou (202102070001), Foshan Engineering Technology Research Center for Drug Safety Re‐evaluation and Clinical Rational Use and the Foshan “14th Five‐Year” Key Medical Specialty Construction Project (FSZD145035), Foshan Self‐funded Science and Technology Innovation Project (2520001002870), and Youth S&T Talent Support Programme of Guangdong Provincial Association for Science and Technology (SKXRC2026308).

## Ethics Statement

All animal experiments were approved by the Institutional Animal Care and Use Committee of Jinan University (Approval No. IACUC‐20240718‐02) and were performed in accordance with the institutional guidelines for animal care and use. Both male and female mice were used in this study.

## Conflicts of Interest

The authors declare no conflicts of interest.

## Supporting information




**Supporting File**: advs78426‐sup‐0001‐SuppMat.docx.

## Data Availability

The data that support the findings of this study are available from the corresponding author upon reasonable request.

## References

[1] C. G. Silva , E. Peyre , and L. Nguyen , “Cell Migration Promotes Dynamic Cellular Interactions to Control Cerebral Cortex Morphogenesis,” Nature Reviews Neuroscience 20, no. 6 (2019): 318–329, 10.1038/s41583-019-0148-y. 30874623

[2] M. G. Andrews , L. Subramanian , J. Salma , and A. R. Kriegstein , “How Mechanisms of Stem Cell Polarity Shape the Human Cerebral Cortex,” Nature Reviews Neuroscience 23, no. 12 (2022): 711–724, 10.1038/s41583-022-00631-3. PMC10571506 36180551

[3] P. Vanderhaeghen and F. Polleux , “Developmental Mechanisms Underlying the Evolution of Human Cortical Circuits,” Nature Reviews Neuroscience 24, no. 4 (2023): 213–232, 10.1038/s41583-023-00675-z. PMC10064077 36792753

[4] S. T. Schafer , A. C. M. Paquola , S. Stern , et al., “Pathological Priming Causes Developmental Gene Network Heterochronicity in Autistic Subject‐Derived Neurons,” Nature Neuroscience 22, no. 2 (2019): 243–255, 10.1038/s41593-018-0295-x. PMC6402576 30617258

[5] I. Parenti , L. G. Rabaneda , H. Schoen , and G. Novarino , “Neurodevelopmental Disorders: From Genetics to Functional Pathways,” Trends in Neurosciences 43, no. 8 (2020): 608–621, 10.1016/j.tins.2020.05.004. 32507511

[6] A. Albajara Sáenz , T. Villemonteix , P. V. Schuerbeek , et al., “Motor Abnormalities in Attention‐Deficit/Hyperactivity Disorder and Autism Spectrum Disorder Are Associated with Regional Grey Matter Volumes,” Frontiers in Neurology 12 (2021): 666980, 10.3389/fneur.2021.666980. PMC8129495 34017307

[7] J. G. Zwicker , M. Suto , S. R. Harris , N. Vlasakova , and C. Missiuna , “Developmental Coordination Disorder Is More Than a Motor Problem: Children Describe the Impact of Daily Struggles on Their Quality of Life,” British Journal of Occupational Therapy 81, no. 2 (2018): 65–73, 10.1177/0308022617735046.

[8] S. Gunasekaran , Y. Miyagawa , and K. Miyamoto , “Actin Nucleoskeleton in Embryonic Development and Cellular Differentiation,” Current Opinion in Cell Biology 76 (2022): 102100, 10.1016/j.ceb.2022.102100. 35605340

[9] D. P. Meka , R. Scharrenberg , B. Zhao , et al., “Radial Somatic F‐Actin Organization Affects Growth Cone Dynamics During Early Neuronal Development,” The EMBO Reports 20, no. 12 (2019): 47743, 10.15252/embr.201947743. PMC6893363 31650708

[10] Z. Zhang , H. Zhang , A. Antonic‐Baker , P. Kwan , Y. Yan , and Y. Ma , “CXCR5 Regulates Neuronal Polarity Development and Migration in the Embryonic Stage via F‐Actin Homeostasis and Results in Epilepsy‐Related Behavior,” Neuroscience Bulletin 39, no. 11 (2023): 1605–1622, 10.1007/s12264-023-01087-w. PMC10603003 37460877

[11] A. M. Diab , M. Wigerius , D. P. Quinn , et al., “NCK1 Modulates Neuronal Actin Dynamics and Promotes Dendritic Spine, Synapse, and Memory Formation,” The Journal of Neuroscience 43, no. 6 (2023): 885–901, 10.1523/jneurosci.0495-21.2022. PMC9908320 36535770

[12] V. Mercaldo , B. Vidimova , D. Gastaldo , et al., “Altered Striatal Actin Dynamics Drives Behavioral Inflexibility in a Mouse Model of Fragile X Syndrome,” Neuron 111, no. 11 (2023): 1760–1775.e8, 10.1016/j.neuron.2023.03.008. 36996810

[13] E. Dazzo , K. Rehberg , R. Michelucci , et al., “Mutations in MICAL‐1 Cause Autosomal‐Dominant Lateral Temporal Epilepsy,” Annals of Neurology 83, no. 3 (2018): 483–493, 10.1002/ana.25167. 29394500

[14] D. Guo , X. Yang , and L. Shi , “Rho GTPase Regulators and Effectors in Autism Spectrum Disorders: Animal Models and Insights for Therapeutics,” Cells 9, no. 4 (2020): 835, 10.3390/cells9040835. PMC7226772 32244264

[15] V. Zamboni , R. Jones , A. Umbach , et al., “Rho GTPases in Intellectual Disability: From Genetics to Therapeutic Opportunities,” International Journal of Molecular Sciences 19, no. 6 (2018): 1821, 10.3390/ijms19061821. PMC6032284 29925821

[16] J. G. Duman , F. A. Blanco , C. A. Cronkite , et al., “Rac‐maninoff and Rho‐vel: The Symphony of Rho‐GTPase Signaling at Excitatory Synapses,” Small GTPases 13, no. 1 (2022): 14–47, 10.1080/21541248.2021.1885264. PMC9707551 33955328

[17] L. K. Nguyen , B. N. Kholodenko , and A. Kriegsheim , “Rac1 and RhoA: Networks, Loops and Bistability,” Small GTPases 9, no. 4 (2018): 316–321, 10.1080/21541248.2016.1224399. PMC5997137 27533896

[18] K. M. Byrne , N. Monsefi , J. C. Dawson , et al., “Bistability in the Rac1, PAK, and RhoA Signaling Network Drives Actin Cytoskeleton Dynamics and Cell Motility Switches,” Cell Systems 2, no. 1 (2016): 38–48, 10.1016/j.cels.2016.01.003. PMC4802415 27136688

[19] M. R. F. Reijnders , N. M. Ansor , M. Kousi , et al., “RAC1 Missense Mutations in Developmental Disorders With Diverse Phenotypes,” The American Journal of Human Genetics 101, no. 3 (2017): 466–477, 10.1016/j.ajhg.2017.08.007. PMC5591022 28886345

[20] S. Banka , A. Bennington , M. J. Baker , et al., “Activating RAC1 Variants in the Switch II Region Cause a Developmental Syndrome and Alter Neuronal Morphology,” Brain 145, no. 12 (2022): 4232–4245, 10.1093/brain/awac049. PMC9762944 35139179

[21] M. Scala , M. Nishikawa , H. Ito , et al., “Variant‐specific Changes in RAC3 Function Disrupt Corticogenesis in Neurodevelopmental Phenotypes,” Brain 145, no. 9 (2022): 3308–3327, 10.1093/brain/awac106. PMC9473360 35851598

[22] F. L. Harms , K. Kloth , A. Bley , et al., “Activating Mutations in PAK1, Encoding p21‐Activated Kinase 1, Cause a Neurodevelopmental Disorder,” The American Journal of Human Genetics 103, no. 4 (2018): 579–591, 10.1016/j.ajhg.2018.09.005. PMC6174322 30290153

[23] S. Horn , M. Au , L. Basel‐Salmon , et al., “De Novo Variants in PAK1 Lead to Intellectual Disability With Macrocephaly and Seizures,” Brain 142, no. 11 (2019): 3351–3359, 10.1093/brain/awz264. PMC6821231 31504246

[24] S. Ohori , S. Mitsuhashi , R. Ben‐Haim , et al., “A Novel PAK1 Variant Causative of Neurodevelopmental Disorder With Postnatal Macrocephaly,” Journal of human genetics 65, no. 5 (2020): 481–485, 10.1038/s10038-020-0728-8. 32005903

[25] A. Hayashi‐Takagi , Y. Araki , M. Nakamura , et al., “PAKs Inhibitors Ameliorate Schizophrenia‐Associated Dendritic Spine Deterioration in Vitro and in Vivo During Late Adolescence,” Proceedings of the National Academy of Sciences 111, no. 17 (2014): 6461–6466, 10.1073/pnas.1321109111. PMC4035976 24706880

[26] A. I. Molosh , P. L. Johnson , J. P. Spence , et al., “Social Learning and Amygdala Disruptions in Nf1 Mice Are Rescued by Blocking p21‐Activated Kinase,” Nature Neuroscience 17, no. 11 (2014): 1583–1590, 10.1038/nn.3822. PMC4213300 25242307

[27] A. Pyronneau , Q. He , J.‐Y. Hwang , M. Porch , A. Contractor , and R. S. Zukin , “Aberrant Rac1‐cofilin Signaling Mediates Defects in Dendritic Spines, Synaptic Function, and Sensory Perception in Fragile X Syndrome,” Science signaling 10, no. 504 (2017): aan0852, 10.1126/scisignal.aan0852. PMC5988355 29114038

[28] B. M. Dolan , S. G. Duron , D. A. Campbell , et al., “Rescue of Fragile X Syndrome Phenotypes in Fmr1 KO Mice by the Small‐Molecule PAK Inhibitor FRAX486,” Proceedings of the National Academy of Sciences 110, no. 14 (2013): 5671–5676, 10.1073/pnas.1219383110. PMC3619302 23509247

[29] K. Ohashi , A. Kunitomi , S. Chiba , and K. Mizuno , “Roles of the Dbl Family of RhoGEFs in Mechanotransduction—A Review,” Frontiers in Cell and Developmental Biology 12 (2024): 1485725, 10.3389/fcell.2024.1485725. PMC11521908 39479515

[30] P. M. Müller , J. Rademacher , R. D. Bagshaw , et al., “Systems Analysis of RhoGEF and RhoGAP Regulatory Proteins Reveals Spatially Organized RAC1 Signalling From Integrin Adhesions,” Nature Cell Biology 22, no. 4 (2020): 498–511, 10.1038/s41556-020-0488-x. 32203420

[31] X. Xiang , S. Li , X. Zhuang , and L. Shi , “Arhgef1 negatively Regulates Neurite Outgrowth Through Activation of RhoA Signaling Pathways,” FEBS Letters 590, no. 17 (2016): 2940–2955, 10.1002/1873-3468.12339. 27489999

[32] X. Xiang , X. Zhuang , S. Li , and L. Shi , “Arhgef1 is Expressed in Cortical Neural Progenitor Cells and Regulates Neurite Outgrowth of Newly Differentiated Neurons,” Neuroscience Letters 638 (2017): 27–34, 10.1016/j.neulet.2016.11.067. 27923664

[33] X. Xiang , X. Jiang , and X. Lu , “Regulation of Neural Stem Cell Self‐Renewal, Proliferation and Differentiation by the RhoA Guanine Nucleotide Exchange Factor Arhgef 1,” Gene 863 (2023): 147306, 10.1016/j.gene.2023.147306. 36813057

[34] M. Jaiswal , L. Gremer , R. Dvorsky , et al., “Mechanistic Insights Into Specificity, Activity, and Regulatory Elements of the Regulator of G‐Protein Signaling (RGS)‐Containing Rho‐specific Guanine Nucleotide Exchange Factors (GEFs) p115, PDZ‐RhoGEF (PRG), and Leukemia‐Associated RhoGEF (LARG),” Journal of Biological Chemistry 286, no. 20 (2011): 18202–18212, 10.1074/jbc.M111.226431. PMC3093892 21454492

[35] C. Li , J. Wei , and D. Wang , “Planar Cell Polarity Protein Celsr2 Maintains Structural and Functional Integrity of Adult Cortical synapses,” Progress in Neurobiology (2022), 102352, 10.1016/j.pneurobio.2022.102352. 36089108

[36] H. C. Wang and D. E. Feldman , “Degraded Tactile Coding in the Cntnap2 Mouse Model of Autism,” Cell reports 43 (2024): 114612, 10.1016/j.celrep.2024.114612. PMC11396660 39110592

[37] A. A. Bhaskaran , T. Gauvrit , Y. Vyas , G. Bony , M. Ginger , and A. Frick , “Endogenous Noise of Neocortical Neurons Correlates With Atypical Sensory Response Variability in the Fmr1−/y Mouse Model of Autism,” Nature Communications 14, no. 1 (2023): 7905, 10.1038/s41467-023-43777-z. PMC10689491 38036566

[38] E. Wilson , T. Rudisill , B. Kirk , C. Johnson , P. Kemper , and K. Newell‐Litwa , “Cytoskeletal Regulation of Synaptogenesis in a Model of Human Fetal Brain Development,” Journal of neuroscience research 98, no. 11 (2020): 2148–2165, 10.1002/jnr.24692. 32713041

[39] M. Scala , M. Nishikawa , K. I. Nagata , and P. Striano , “Pathophysiological Mechanisms in Neurodevelopmental Disorders Caused by Rac GTPases Dysregulation: What's Behind Neuro‐RACopathies,” Cells 10, no. 12 (2021): 3395, 10.3390/cells10123395. PMC8699292 34943902

[40] H. Rosenfeldt , M. D. Castellone , P. A. Randazzo , and J. S. Gutkind , “Rac Inhibits Thrombin‐Induced Rho Activation: Evidence of a Pak‐dependent GTPase Crosstalk,” Journal of Molecular Signaling 1 (2006): 8, 10.1186/1750-2187-1-8. PMC1769496 17224083

[41] T. Sarowar , S. Grabrucker , K. Föhr , et al., “Enlarged Dendritic Spines and Pronounced Neophobia in Mice Lacking the PSD Protein RICH2,” Molecular Brain 9, no. 1 (2016): 28, 10.1186/s13041-016-0206-6. PMC4788860 26969129

[42] C. Quevedo , V. Sauzeau , M. Menacho‐Marquez , A. Castro‐Castro , and X. R. Bustelo , “Vav3‐Deficient Mice Exhibit a Transient Delay in Cerebellar Development,” Molecular Biology of the Cell 21, no. 6 (2010): 1125–1139, 10.1091/mbc.e09-04-0292. PMC2836963 20089829

[43] S. Donald , T. Humby , I. Fyfe , et al., “P‐Rex2 Regulates Purkinje Cell Dendrite Morphology and Motor Coordination,” Proceedings of the National Academy of Sciences 105, no. 11 (2008): 4483–4488, 10.1073/pnas.0712324105. PMC2393786 18334636

[44] B. L. Johnson‐Kerner , A. Garcia Diaz , S. Ekins , and H. Wichterle , “Kelch Domain of Gigaxonin Interacts With Intermediate Filament Proteins Affected in Giant Axonal Neuropathy,” PLoS ONE 10, no. 10 (2015): 0140157, 10.1371/journal.pone.0140157. PMC4604155 26460568

[45] L. Civiero and E. Greggio , “PAKs in the Brain: Function and Dysfunction,” Biochimica et Biophysica Acta (BBA)—Molecular Basis of Disease 1864, no. 2 (2018): 444–453, 10.1016/j.bbadis.2017.11.005. 29129728

[46] Y. Guo , C. Benson , M. Hill , et al., “Therapeutic Potential of Pak1 Inhibition for Pain Associated With Cutaneous Burn Injury,” Molecular Pain 14 (2018): 1744806918788648, 10.1177/1744806918788648. PMC6053256 29956587

[47] A. Rubtsov , P. Strauch , A. DiGiacomo , J. Hu , R. Pelanda , and R. M. Torres , “Lsc Regulates Marginal‐Zone B Cell Migration and Adhesion and Is Required for the IgM T‐Dependent Antibody Response,” Immunity 23, no. 5 (2005): 527–538, 10.1016/j.immuni.2005.09.018. 16286020

[48] D. Guo , Y. Peng , L. Wang , et al., “Autism‐Like Social Deficit Generated by Dock4 Deficiency Is Rescued by Restoration of Rac1 Activity and NMDA Receptor Function,” Molecular Psychiatry 26, no. 5 (2021): 1505–1519, 10.1038/s41380-019-0472-7. PMC8159750 31388105

[49] X. Ma , L. Li , Z. Li , et al., “eEF2 in the Prefrontal Cortex Promotes Excitatory Synaptic Transmission and Social Novelty Behavior,” The EMBO Reports 23, no. 10 (2022): 54543, 10.15252/embr.202154543. PMC9535807 35993189

[50] L. Shi , B. Butt , F. C. F. Ip , et al., “Ephexin1 is Required for Structural Maturation and Neurotransmission at the Neuromuscular Junction,” Neuron 65, no. 2 (2010): 204–216, 10.1016/j.neuron.2010.01.012. PMC3718047 20152127

[51] Y. Xiao , Y. Peng , J. Wan , et al., “The atypical Guanine Nucleotide Exchange Factor Dock4 Regulates Neurite Differentiation Through Modulation of Rac1 GTPase and Actin Dynamics,” Journal of Biological Chemistry 288, no. 27 (2013): 20034–20045, 10.1074/jbc.M113.458612. PMC3707701 23720743

[52] M. Huang , H. Ye , Y. Xu , et al., “Correction of eIF4E Overactivation Rescues Translatome Imbalance and Core ASD‐Like Behaviors in Valproic Acid‐Induced Offspring Mice,” Molecular Psychiatry 31, no. 7 (2026): 3987–4005, 10.1038/s41380-026-03517-3. PMC13269136 41795048

[53] X. Yang , D. Guo , K. Li , and L. Shi , “Altered Postnatal Developmental Patterns of Ultrasonic Vocalizations in Dock4 Knockout Mice,” Behavioural Brain Research 406 (2021): 113232, 10.1016/j.bbr.2021.113232. 33705839

