# Supplementary material for: Arhgef1 Deletion Disrupts Early Cortical Neurogenesis via PAK1 Overactivation and Causes Motor Dysfunction in Adulthood
Source: Adv Sci (Weinh). 2026 Oct 25:e78426. Online ahead of print. doi: 10.1002/advs.78426 (PMC13696771; doi:10.1002/advs.78426)
Supplement: Supplementary file 1 [file ADVS-9999-e78426-s001.docx]

***Arhgef1* deletion disrupts early cortical neurogenesis via PAK1 overactivation and causes motor dysfunction in adulthood**

Liuren Li, Xuanyue Ma, Zhenyu Ding, Bang Sun, Linglin Liu, Hei Matthew Yip, Wei Wang, Tianying Wu, Yuan Guo, Yinghui Peng, Shiqing Zhang, Jacque Pak Kan Ip, Li Zhang, Keshen Li, Lei Shi

**Supplementary Figures**

**Supplementary Figure 1. ARHGEF1 expression in different developmental stages and different cell types. a, b** *Arhgef1* mRNA expression levels in cortex (**a**) and hippocampus (**b**) from E12.5 or E16.5 to P60. n = 3–4 mice at each stage. E, embryonic day; P, postnatal day; HP, hippocampus. **c** Immunofluorescent staining of ARHGEF1 (green) and SOX2 (red) in mouse cortex at E13.5. Scale bar, 20 μm. **d** Percentage of SOX2^+^ or SOX2^-^ cells in ARHGEF1^+^ cells were quantified. n = 3 mice. **e** Immunofluorescent staining of ARHGEF1 (green) and different brain cell types (red: NeuN/GFAP/IBA1/OLIG2) in the somatosensory cortex of adult mice. Arrows indicate highly colocalized cells. Scale bar, 10 μm. **f** Colocalization of ARHGEF1 with each cellular marker in (**e**) was quantified by calculating the Manders’ colocalization coefficients. n = 3 mice in each condition. One-way ANOVA with Bonferroni’s multiple comparison test, ***P* = 0.0079 (NeuN^+^/ARHGEF1^+^ *vs.* GFAP^+^/ARHGEF1^+^), ****P* = 0.0002 (NeuN^+^/ARHGEF1^+^ *vs.* IBA1^+^/ARHGEF1^+^), *P* = 0.0513 (NeuN^+^/ARHGEF1^+^ *vs.* OLIG2^+^/ARHGEF1^+^). **g** Immunofluorescent staining of ARHGEF1 (green) and different neuronal markers (red: CaMKIIα or GAD65/67) in the somatosensory cortex of adult mice. Arrows indicate highly colocalized cells. Scale bar, 10 μm. **h** Colocalization of ARHGEF1 and neuron markers in (**g**) was quantified by calculating the Manders’ colocalization coefficients. n= 3 mice in each condition. Unpaired *t* test, **P* = 0.0358. n.s., no significant difference. All data are shown as mean ± SEM.

**Supplementary Figure 2. Characterization of *Arhgef1* cKO mice. a** Genotypes of WT and cKO mice were determined by PCR using both Loxp-F/R and *Emx1*-*Cre*-F/R primers. **b** *Arhgef1* mRNA expression levels in adult cerebellum and thalamus of WT and cKO mice. n = 3 mice per group. Unpaired *t* tests, cerebellum, *P* = 0.6525; thalamus, *P* = 0.8572. **c** Representative Western blots of ARHGEF1 expressions in adult cerebellum and thalamus of WT and cKO mice. **d** Quantification analysis of (**c**). β-actin serves as a loading control. Unpaired *t* test. Cerebellum, *P* = 0.8985, n = 3 mice per group; thalamus, *P* = 0.6455, n = 4 mice per group. **e** Birth rate of WT and cKO mice. **f**, **g** Body weight of male (**f**) and female (**g**) WT and cKO littermates from postnatal week 1 to 11. Two-way ANOVA with Bonferroni’s multiple comparison test. Male, *P* = 0.2054 (WT, n = 18; cKO, n = 16); female, *P* = 0.1342 (WT, n = 16; cKO, n = 14). **h** Brain weight of male and female adult WT and cKO mice. n = 3 mice per group. Unpaired *t* test. Male, *P* = 0.2664; female, *P* = 0.8704. **i** Immunofluorescent staining of NeuN (red) in hippocampal CA1 and CA3 regions of adult WT and cKO mice. Scale bar, 10 μm. **j, k** Number of NeuN^+^ cells per mm^2^ in CA1 (**j**) and CA3 (**k**). n = 3 mice per group. Unpaired *t* test, CA1, *P* = 0.2642; CA3, *P* = 0.8922. n.s., no significant difference. All data are shown as mean ± SEM.

**Supplementary Figure 3. Increased F-actin level in the cortex of *Arhgef1* cKO mice at E13.5. a** Representative cortical sections stained with phalloidin (red) and DAPI (blue) from WT and cKO mice at E13.5. Layers are identified by DAPI-stained nuclear distribution pattern. MZ, marginal zone. CP, cortical plate. IZ, intermediate zone. SVZ, subventricular zone. VZ, ventricular zone. **b** Quantification of fluorescent intensity of phalloidin (fold of WT). Unpaired *t* tests, WT, n = 8 slices from 3 mice, cKO, n = 9 slices from 3 mice, **P* = 0.0209. **c** Representative cortical sections of WT and cKO mice immunostained for p-PAK1 (green) and DAPI (blue) at E16.5. SP, subplate. **d** Number of p-PAK1^+^ cells per mm^2^ in different layers from WT and cKO cortex. n = 6 slices from 3 mice in each group. Unpaired *t* tests, MZ, ****P* = 0.0003; CP, ***P* = 0.005; SP, *****P* < 0.0001; IZ, ****P* = 0.0003; SVZ/VZ, *P* = 0.8105. **e** Mean fluorescent intensity of p-PAK1 in different Layers from WT and cKO cortex. n = 6 slices from 3 mice in each group. Unpaired *t* tests, MZ, ****P* = 0.0001; CP, *****P* < 0.0001; SP, ****P* = 0.0004; IZ, ***P* = 0.001; SVZ/VZ, *P* = 0.5633. All data are shown as mean ± SEM.

**Supplementary Figure 4. PAK1-Cofilin signaling in the hippocampus of WT and *Arhgef1* cKO mice. a** Representative Western blots showing GTP-bound and total protein levels of RHOA, RAC1 and CDC42 in the hippocampus of P6 and adult WT and cKO mice. HP, hippocampus. β-actin serves as a loading control. **b** Quantification of GTP-RHOA levels that were normalized to total-RHOA. Unpaired *t* tests, P6, **P* = 0.0337 (WT, n = 4 mice, cKO, n = 3 mice); Adult, *P* = 0.4494, n = 5 mice per group. **c** Quantification of GTP-RAC1 levels that were normalized to total-RAC1. Unpaired *t* tests, P6, *P* = 0.3436, n = 3 mice per group; Adult, **P* = 0.0212, n = 3 mice per group. **d** Quantification of GTP-CDC42 levels that were normalized to total-CDC42. Unpaired *t* tests, P6, *P* = 0.5525, n = 3 mice per group; Adult, *P* = 0.5362, n = 3 mice per group. n.s., no significant difference. All data are shown as mean ± SEM.

**Supplementary Figure 5. Unaltered behaviors in adult *Arhgef1* cKO mice. a** Duration spent in left and right chambers by WT and cKO mice during habituation in Three-chamber test. Unpaired *t* tests, WT, *P* = 0.7031; cKO, *P* = 0.5382. **b** Time spent on sniffing a social conspecific (S1) and an empty cup (E) by WT and cKO mice during social approach session in Three-chamber test. Unpaired *t* tests, *****P* < 0.0001. **c** Time spent on sniffing a familiar mouse (S1) and an unfamiliar mouse (S2) by WT and cKO mice during social novelty session in Three-chamber test. Unpaired *t* tests, **P* = 0.0157 (WT), ***P* = 0.0021 (cKO). **d** Time spent on sniffing a familiar mouse (S1) and another unfamiliar mouse (S3) by WT and cKO mice during social memory session in Three-chamber test. Unpaired *t* tests, **P* = 0.0154 (WT), *****P* < 0.0001 (cKO). WT, n = 11 mice; cKO, n = 11 mice. **e** Total call duration during 5-min recording of separation-induced ultrasonic vocalizations (USVs) by WT and cKO pups at different postnatal days (P3, P6, P9, P12). Two-way ANOVA with Bonferroni’s multiple comparison test, *P* = 0.4491. **f** Number of calls by WT and cKO pups at P3, P6, P9 and P12. Two-way ANOVA with Bonferroni’s multiple comparison test, *P* = 0.8550. WT, n = 14 pups; cKO, n = 20 pups (**e**, **f**). **g** Time spent on grooming by WT and cKO mice during 20 min test. WT, n = 11 mice; cKO, n = 10 mice. Mann-Whitney tests, *P* = 0.1321. **h** Number of marbles buried by WT and cKO mice. WT, n = 14 mice; cKO, n = 18 mice. Unpaired *t* tests, *P* = 0.7031. **i** Time spent on sniffing two identical objects by WT and cKO mice in the training phase of Novel object recognition test. Mann-Whitney test, *P* = 0.7477 (left); *P* = 0.7394 (right). **j** The preference (% of time sniffing) for familiar object (F) or novel object (N) of WT and cKO mice in the test phase 1 h after training. Unpaired *t* tests, *****P* < 0.0001 (WT); **P* = 0.0145 (cKO). **k** The preference for F or N 24 h after training. Unpaired *t* tests, ***P* = 0.0063 (WT); ***P* = 0.0011 (cKO). i–k, WT, n = 11 mice; cKO, n = 10 mice. **l** The alternation of WT and cKO mice in Y-maze test. An alternation was defined as a nonoverlapping entrance sequence in three arms. WT, n = 19 mice; cKO, n = 24 mice. Unpaired *t* tests, *P* = 0.2524. **m** Number of arm entries by WT and cKO mice in Y-maze test. Unpaired *t* tests, *P* = 0.7446. **n** Forepaw and hindpaw stride length of WT and cKO mice. Stride length was calculated as the distance between two consecutive footprints of the same paw. Unpaired *t* tests, forepaw, *P* = 0.6193; hindpaw, *P* = 0.7035. **o** Forepaw and hindpaw base width of WT and cKO mice. Base width was measure as the distance between the paired footprints of the two forepaws or two hindpaws. Unpaired *t* tests, forepaw, *P* = 0.2003; hindpaw, *P* = 0.7236. **p** Left and right overlap of WT and cKO mice. Overlap was defined as the distance between the paired footprints of the forepaws and hindpaws from the same body side. Unpaired *t* tests, left, *P* = 0.5057; right, *P* = 0.9172. n–p, WT, n = 14 mice; cKO, n = 13 mice. n.s., no significant difference. All data are shown as mean ± SEM.

**Supplementary Figure 6. Persistent motor deficits in 15-month-old *Arhgef1* cKO mice. a**, **b** Travel distance (**a**) and duration in center (**b**) of aged WT and cKO mice in Open-field test. WT, n = 12 mice; cKO, n = 19 mice. Unpaired *t* tests, *P* = 0.3894 (**a**); *P* = 0.1445 (**b**). **c** Alternation of aged WT and cKO mice in Y-maze test. Unpaired *t* test, *P* = 0.8729. **d** Number of arm entries by aged WT and cKO mice in Y-maze test. Mann Whitney test. *P* = 0.8013. WT, n = 12 mice; cKO, n = 19 mice (**c**, **d**). **e** Duration in open section by aged WT and cKO mice in Elevated Zero-maze. Mann-Whitney test, *P* =0.6675. **f** Number of head dips made by aged WT and cKO mice in open sections. Mann-Whitney test, *P* = 0.7565. WT, n = 12 mice; cKO, n = 19 mice. **g** Latency to fall in individual trials across 3 test days of aged WT and cKO mice in accelerating rotarod test. WT, n = 12 mice; cKO, n = 19 mice. Two-way ANOVA with Bonferroni’s multiple comparison post hoc, ****P* = 0.0004. n.s., no significant difference. All data are shown as mean ± SEM.

**Supplementary Materials and Methods**

**Genotyping**

Genotyping was performed via PCR using genomic DNA extracted from mice tails. The non-floxed and floxed allele of *Arhgef1* were identified by PCR products of 550 bp and 250 bp, respectively, amplified using the 5’ A1-Loxp primer and 3’ A1-Loxp primer. Heterozygous mice (*Arhgef1^fl/+^*) were verified by the presence of both 550 bp and 250 bp products. *Emx1*-*Cre* expression was confirmed by a 700 bp PCR product using Cre-F and Cre-R primers, while mice lacking *Emx1*-*Cre* showed no PCR products. *Arhgef1^fl/fl^* mice with *Emx1*-*Cre* expression were designated as *Arhgef1* cKO mice.

The primer sequences are as follows (from 5’ to 3’):

A1-Loxp Primer-F: AGTCCCGGATTCATATTGAAG

A1-Loxp Primer-R: TCCTAAGCTCAATGGAGCCTC

Cre-F: ATCCGAAAAGAAAACGTTGA

Cre-R: ATCCAGGTTACGGATATAGT

**Grooming analysis**

Grooming was evaluated in a new home cage with fresh beddings. Mice were habituated in the cage for 20 min, and grooming behaviors in the next 20 min were counted.

**Marble-burying**

Marble-burying test was conducted in a box (31.5 cm × 20.5 cm × 18 cm) with 4-cm-thickness beddings. 20 pieces of black marbles (diameter 1.5 cm) were placed on top of the beddings in a 4 × 5 array with a spacing of 3 cm between each marble. Mouse was gently placed in the cage and allowed to freely behave for 20 min. The number of buried marbles, defined as over two-thirds of marble surface area was buried, was counted.

**Three-chamber test**

This test was performed as previously described ^1, 2^. In brief, this test consists of four phases. Firstly, in habituation phase, the test mouse was allowed to explore three chambers freely for 10 min. In social approach phase, an empty wire cup and a wire cup with an unfamiliar mouse (Stranger 1) inside were placed in each of the side chambers respectively. And the test mouse was allowed to explore three chambers for 10 min. In social novelty phase, a novel mouse (Stranger 2) was placed in the empty wire cup and the test mouse was allowed to explore for 10 min. Then both Stranger 1 and 2 were removed from cups, leaving the test mouse to explore for 10 min. In social memory phase, Stranger 1 and another novel mouse (Stranger 3) were placed in each of the side chambers respectively, and the test mouse was allowed to explore for 10 min. The time spent in each chamber and on sniffing empty cups or strangers were calculated by Topscan 3.0 (Clever Sys Inc). All mice in the cups were age- and gender-matched with the test mouse.

**Ultrasonic vocalization recording**

Calls of isolated pups on postnatal day 3, 6, 9, 12 were recorded through Avisoft UltraSound Gate 116 Hm (Avisoft Bioacoustics) as described before ^3^. In brief, every pup was separated from dams and littermates, and laid on a glass beaker with new bedding. And the beaker was placed in a soundproof box at 20℃ which was a cold stimulation for pups. A high-quality condenser microphone was used to record ultrasounds of pups for 5 mins. Finally, pups were returned to homecages and back to their dams and littermates. The total call duration and number of calls were analyzed through Avisoft SASLab Pro (version 4.2, Avisoft Bioacoustics).

**Y-maze working memory**

Spontaneous alteration test was performed as previously described ^1, 2^. Each mouse was placed at the start of one of three arms randomly to switch and explore in three arms freely for 8 min. A successful alternation was defined as a nonoverlapping entrance sequence in three arms. The percentage of alternation and number of entrances to all arms were calculated as referred^1^.

**Novel object recognition**

Novel object recognition was conducted as described before ^1, 2^. Briefly, mice were first habituated in the Open-field arena for 10 min. After 1 h, two same white bottle caps were sticked to the bottom of box with a distance of 30 cm apart. And mice were allowed to recognize the two same objects for 10 min. After mice back to homecages for 1 h, one of the white bottle caps was replaced by a yellow sphere, and mice were allowed to perform novel-object-recognition phase for 10 min. Mice were returned to homecages following the test, and re-introduced to the arena for another recognition test 24 h after the training session, when the yellow sphere was substituted with a lego block. Topscan 3.0 was used to analyze travel distance and sniffing time around each object.

**Reference**

1.	Ma X*, et al.* eEF2 in the prefrontal cortex promotes excitatory synaptic transmission and social novelty behavior. *EMBO Rep* **23**, e54543 (2022).

2.	Guo D*, et al.* Autism-like social deficit generated by Dock4 deficiency is rescued by restoration of Rac1 activity and NMDA receptor function. *Molecular psychiatry* **26**, 1505-1519 (2021).

3.	Yang X, Guo D, Li K, Shi L. Altered postnatal developmental patterns of ultrasonic vocalizations in Dock4 knockout mice. *Behavioural Brain Research* **406**, 113232 (2021).
